# Outcome Prediction Models for Endovascular Treatment of Ischemic Stroke: Systematic Review and External Validation

**DOI:** 10.1161/STROKEAHA.120.033445

**Published:** 2021-11-04

**Authors:** Femke Kremers, Esmee Venema, Martijne Duvekot, Lonneke Yo, Reinoud Bokkers, Geert Lycklama À. Nijeholt, Adriaan van Es, Aad van der Lugt, Charles Majoie, James Burke, Bob Roozenbeek, Hester Lingsma, Diederik Dippel

**Affiliations:** Neurology, Erasmus Medical Center, Erasmus MC Stroke Center, Rotterdam, the Netherlands (F.K., E.V., M.D., B.R., D.D.).; Public Health, Erasmus Medical Center, Rotterdam, the Netherlands (E.V., H.L.).; Neurology, Albert Schweitzer Hospital, Dordrecht, the Netherlands (M.D.).; Radiology, Catharina Medical Center, Eindhoven, the Netherlands (L.Y.).; Radiology, UMCG Groningen Medical Center, the Netherlands (R.B.).; Radiology, Haaglanden Medical Center, The Hague, the Netherlands (G.L.A.N.).; Radiology, Leiden Medical Center, the Netherlands (A.v.E.).; Radiology, Erasmus Medical Center, Rotterdam, the Netherlands (A.v.d.L.).; Radiology, Amsterdam Medical Center, the Netherlands (C.M.).; Neurology, University of Michigan, Ann Arbor (J.B.).

**Keywords:** calibration, ischemic stroke, population, prognosis, publications, systematic review

## Abstract

**Background and Purpose:**

Prediction models for outcome of patients with acute ischemic stroke who will undergo endovascular treatment have been developed to improve patient management. The aim of the current study is to provide an overview of preintervention models for functional outcome after endovascular treatment and to validate these models with data from daily clinical practice.

**Methods:**

We systematically searched within Medline, Embase, Cochrane, Web of Science, to include prediction models. Models identified from the search were validated in the MR CLEAN (Multicenter Randomized Clinical Trial of Endovascular Treatment for Acute Ischemic Stroke in the Netherlands) registry, which includes all patients treated with endovascular treatment within 6.5 hours after stroke onset in the Netherlands between March 2014 and November 2017. Predictive performance was evaluated according to discrimination (area under the curve) and calibration (slope and intercept of the calibration curve). Good functional outcome was defined as a score of 0–2 or 0–3 on the modified Rankin Scale depending on the model.

**Results:**

After screening 3468 publications, 19 models were included in this validation. Variables included in the models mainly addressed clinical and imaging characteristics at baseline. In the validation cohort of 3156 patients, discriminative performance ranged from 0.61 (SPAN-100 [Stroke Prognostication Using Age and NIH Stroke Scale]) to 0.80 (MR PREDICTS). Best-calibrated models were THRIVE (The Totaled Health Risks in Vascular Events; intercept −0.06 [95% CI, −0.14 to 0.02]; slope 0.84 [95% CI, 0.75–0.95]), THRIVE-c (intercept 0.08 [95% CI, −0.02 to 0.17]; slope 0.71 [95% CI, 0.65–0.77]), Stroke Checkerboard score (intercept −0.05 [95% CI, −0.13 to 0.03]; slope 0.97 [95% CI, 0.88–1.08]), and MR PREDICTS (intercept 0.43 [95% CI, 0.33–0.52]; slope 0.93 [95% CI, 0.85–1.01]).

**Conclusions:**

The THRIVE-c score and MR PREDICTS both showed a good combination of discrimination and calibration and were, therefore, superior in predicting functional outcome for patients with ischemic stroke after endovascular treatment within 6.5 hours. Since models used different predictors and several models had relatively good predictive performance, the decision on which model to use in practice may also depend on simplicity of the model, data availability, and the comparability of the population and setting.

## Abstract 2

Supplemental Digital Content is available in the text.

Some patients with stroke may benefit more from endovascular treatment (EVT) than others, depending on their clinical, radiological, or biological characteristics. In a demanding clinical situation with time constraints, a tool that helps predicting outcome after treatment may guide decision making and may be helpful in providing prognostic information to patient and family.

Multiple prediction models have, therefore, been developed to predict outcome of individual patients treated with EVT. Some of these models have already been externally validated and implemented in clinical care, others still require further validation.^1–4^ However, no single model has emerged as the optimal model for EVT patient selection, in part, because little is known about the comparative performance of existing models.

Therefore, the aim of this study is to provide a systematic review of preintervention prediction models for functional outcome for patients receiving EVT and to externally validate these models with data from patients treated in daily clinical practice.

## Methods

We performed this systematic review in accordance with the Preferred Reporting Items for Systematic Review and Meta-Analysis guidelines (Table I and Supplementary Material I in the Supplemental Material).^5^

### Systematic Literature Search

We conducted a systematic search for studies that reported an outcome prediction model for patients with stroke treated with EVT on May 18, 2020, in the databases Embase, MEDLINE, Cochrane, and Web of Science. The search strategy contained search terms such as “Prediction,” “Thrombectomy,” “Endovascular Therapy,” and “Acute Ischemic Stroke” (Supplementary Material I in the Supplemental Material). The search was restricted to studies published in English, and conference abstracts were excluded. Articles were screened on title and abstract and subsequently assessed for eligibility based on full text by 2 independent reviewers (F. Kremers and M. Duvekot). Discrepancies between authors were discussed until consensus was reached. Data from the MR CLEAN (Multicenter Randomized Clinical Trial of Endovascular Treatment for Acute Ischemic Stroke in the Netherlands) registry cannot be made publicly available, but all statistical analyses and syntax may be provided upon reasonable request.

### Inclusion Criteria

Articles were included when the development of a prediction model or score was the main purpose of the study, or when an existing model for intravenous thrombolysis was validated on patients treated with EVT. Included models had to comply with the following: patients with a proximal arterial occlusion in the anterior cerebral circulation demonstrated by computed tomography angiography or magnetic resonance angiography; predict outcome after thrombectomy independent of device type; consist of at least 2 variables; and consider only variables that can be measured before start of EVT. Assessment of functional outcome had to be done using the modified Rankin Scale (mRS).

### Quality Assessment

We evaluated the prediction models with the Prediction Model Risk of Bias Assessment Tool (PROBAST), which was developed to assess the methodological quality of a prediction model (Supplementary Material II in the Supplemental Material).^6^ The PROBAST questionnaire assesses the risk of bias and applicability of a prediction model in 4 domains: participants, predictors, outcome, and analysis. Two independent reviewers (F. Kremers and E. Venema) conducted the PROBAST evaluation and, in case of a disagreement, a third independent reviewer (J. Burke or D. Dippel) was consulted for adjudication.

### Validation Cohort

External validation was performed with data from the MR CLEAN registry parts I and II. All consecutive patients treated with EVT in the Netherlands were included in the registry, from March 18, 2014, until November 1, 2017. In total, 3156 patients from 17 centers were included. Patients were included in the validation cohort if they were 18 years or older, were treated within 6.5 hours from onset, with a proximal arterial occlusion in the anterior cerebral circulation (internal carotid artery, internal carotid artery terminus, middle cerebral artery M1/M2) confirmed by computed tomography angiography or magnetic resonance angiography.

### Analysis

We analyzed prediction models as intended for clinical use. Simplified risk scores that attributed points for a variable that had been dichotomized or trichotomized were implemented as such in the validation cohort. The predicted probabilities of functional outcome after risk score calculation were compared with the observed probabilities in the validation cohort for patients receiving EVT. The corresponding author was contacted when probabilities were not available. Collateral scores were implemented for validation as graded in the validation cohort.^7^

Discriminative performance was measured with the area under the receiver operator curve (AUC). The discriminative ability of a prediction model indicates the ability to separate the patients with a poor functional outcome from patients with a good functional outcome. A value between 0.7 and 0.8 is generally considered as good discriminative ability, and an AUC higher than 0.8 is considered excellent.^8–10^ AUCs were formally compared for significance with the DeLong test.^11^ Calibration plots were developed to assess the level of agreement between predicted risks and observed outcome.^12,13^ Calibration is a useful and reliable tool for external validation of prediction models since the slope and intercept derived from calibration plot provide an overall estimate of systematic overestimation or underestimation in the validation cohort.^14^ The slope of a model may also be described as the coefficient of the logistic calibration analysis. Ideally, the calibration curve has a slope of 1 and an intercept of 0. In our analysis, we assessed models for the best combination of the slope closest to 1 and the intercept closest to 0.^15^ We selected 5 models with the best intercept and 5 models with the best slope. Models with a combination of a top-5 intercept and slope were defined as best-calibrated models. To summarize the absolute difference between the predicted and the observed probabilities, the average errors (E_avg_) and maximum errors (E_max_) of the prediction models were calculated. The E_avg_ and E_max_ represent the average and maximum error between the predicted probabilities and observed calibrated probabilities of functional outcome.^16^

If a study expressed a probability of 0 (0%) or 1 (100%) for the outcome of interest, this value was adapted to 1% or 99%, since the val.prob.ci.2 function in R otherwise excludes these probabilities for calibration.^13^

Multiple imputation by chained equations based on relevant covariates and outcome variables was implemented to account for missing values in the validation cohort. CIs were composed with bootstrapping (200 samples in 5 imputed datasets) for the AUC and calibration statistics of the model.

All analyses were performed with R Statistical Software 3.6.1 with the following packages: foreign, haven, pROC, mice, shiny, DBI, remotes, rms, devtools, gbm, BavoDC, ggplot2, and forestplot.

## Results

**Figure 1.**
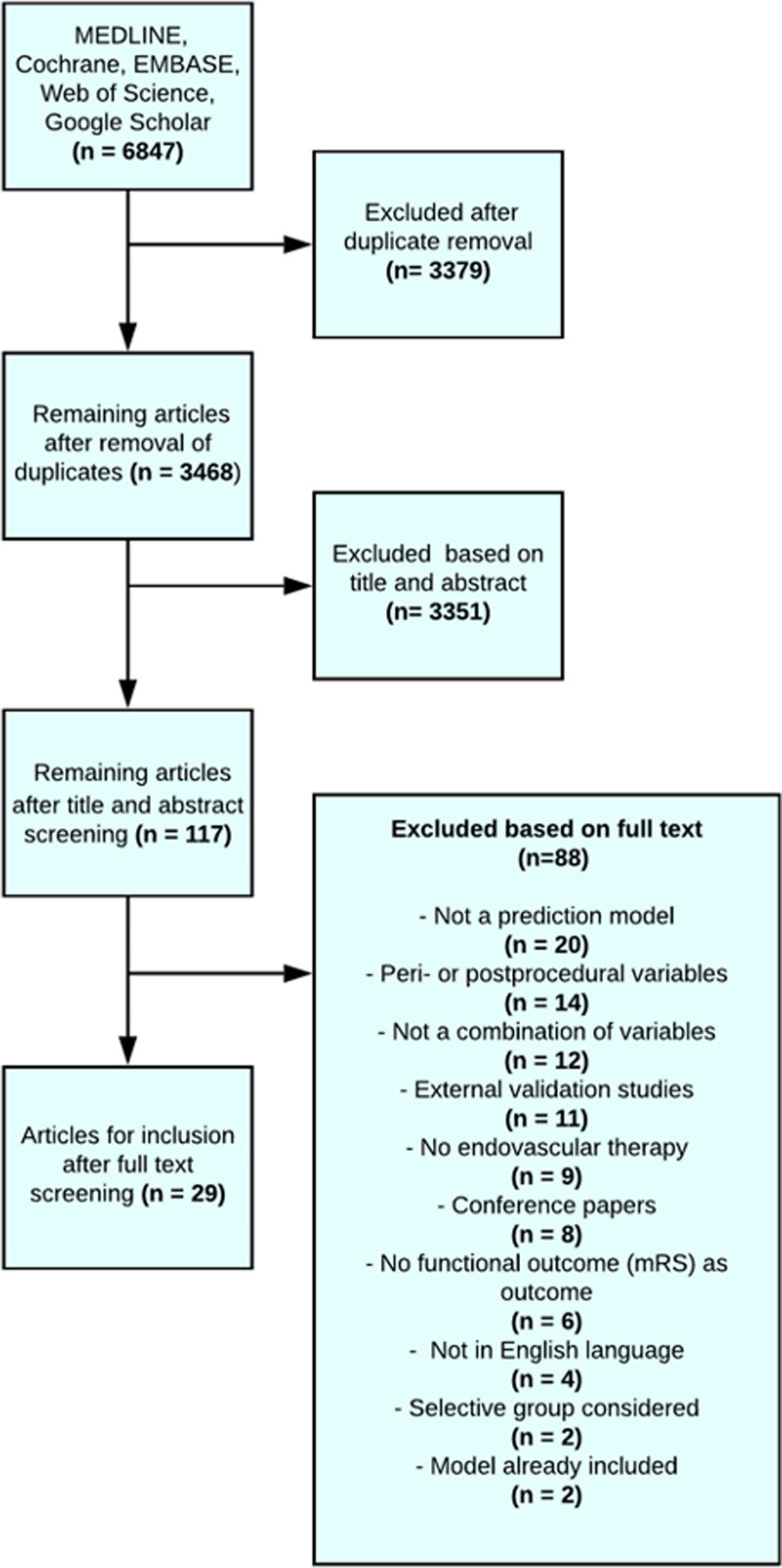
**Flowchart of the selected prediction models after a systematic search of the literature.** mRS indicates modified Rankin Scale.

In total, 3468 articles were identified after the removal of duplicates. After the exclusion of 3351 articles based on title and abstract, 117 articles remained for full-text analysis. Of those, 29 articles describing 31 models were included for further evaluation (Figure 1, Table 1).^17–51^ Four articles used machine learning techniques and could not be included in the PROBAST quality assessment and external validation.^27,33,34,37,50^ Thus, 25 articles containing 27 prediction models could be assessed for PROBAST quality analysis. In total, 17 articles describing 19 models contained variables that were available in the MR CLEAN registry and could be externally validated.The included models used between 2 and 11 predictors, which are listed in Table II in the Supplemental Material.^17–26,28–32,35,36,38–49,51^ Most often used clinical predictors were age and stroke severity measured by the National Institutes of Health Stroke Scale or the Canadian Neurological Score. Computed tomography collateral score and Alberta Stroke Program Early CT Score were the most widely used radiological variables. An overview of models and their calculations are described in Tables III and IV in the Supplemental Material.

**Table 1.**
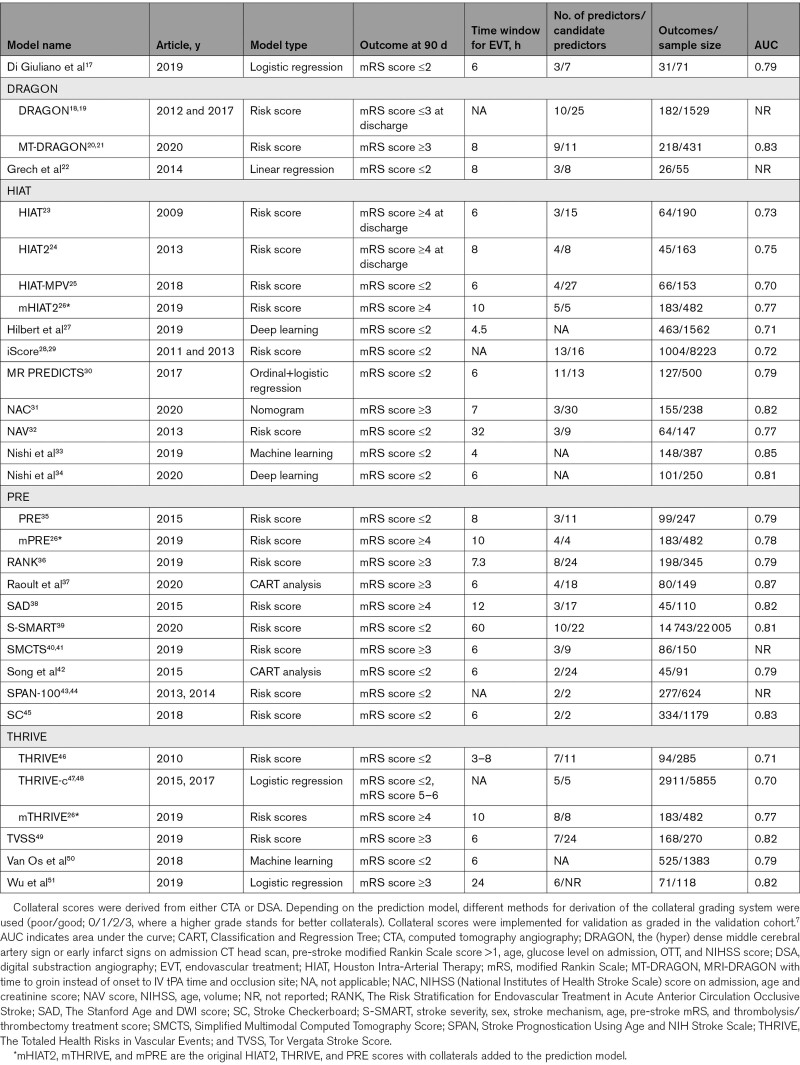
Overview of Prediction Models Included After Systematic Review of the Literature, in Alphabetic Order, With Variants of a Prediction Model Grouped Together

Some variables used in selected prediction models were not available in the validation cohort and could, therefore, not be assessed on predictive performance (Supplementary Material III in the Supplemental Material).

### Quality Assessment

Ten articles (40%) were assessed by a second reviewer, with an interrater reliability of 87% after discussion of discrepancies.

All models had certain methodological shortcomings in their development and were considered at high risk of bias in the domain analysis (Table V in the Supplemental Material, Supplementary Material IV in the Supplemental Material). Models with the best methodological quality according to the PROBAST questionnaire were the MR PREDICTS, S-SMART, and the THRIVE-c (Supplementary Materials II and IV in the Supplemental Material, Table V and Figure I in the Supplemental Materials).

### External Validation

In the validation cohort (n=3156), mean age was 70 years (±14) and median National Institutes of Health Stroke Scale was 16 (interquartile range, 11–19; Table VI in the Supplemental Material). One thousand one hundred ninety-three patients (40.5%) had an mRS score of 0–2, indicating functional independence after 3 months.

**Figure 2.**
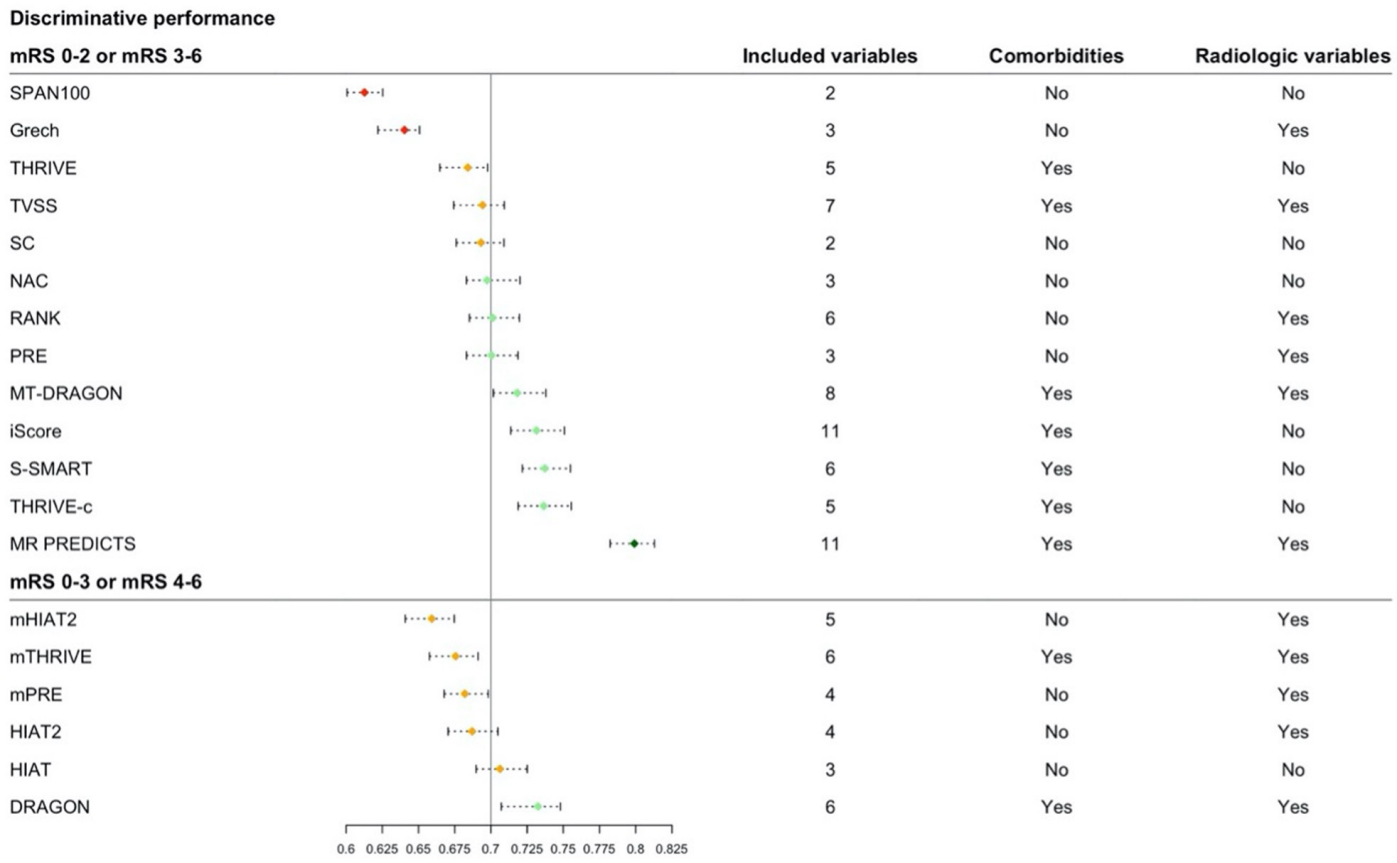
**Overview of the area under the curve (AUC) ranked by discriminative performance for different Rankin Scale score cut points separately.** Red: poor discrimination (AUC, 0.6–0.64), orange: poor discrimination (0.65–0.69), green: acceptable discrimination (0.70–0.79), and dark green: excellent discrimination (0.80 or higher).^8–10^ Per model is described how many variables were in the final model, and whether they included comorbidities and/or radiological variables in their model. mRS indicates modified Rankin Scale; SC, Stroke Checkerboard; and TVSS, Tor Vergata Stroke Score.

**Figure 3.**
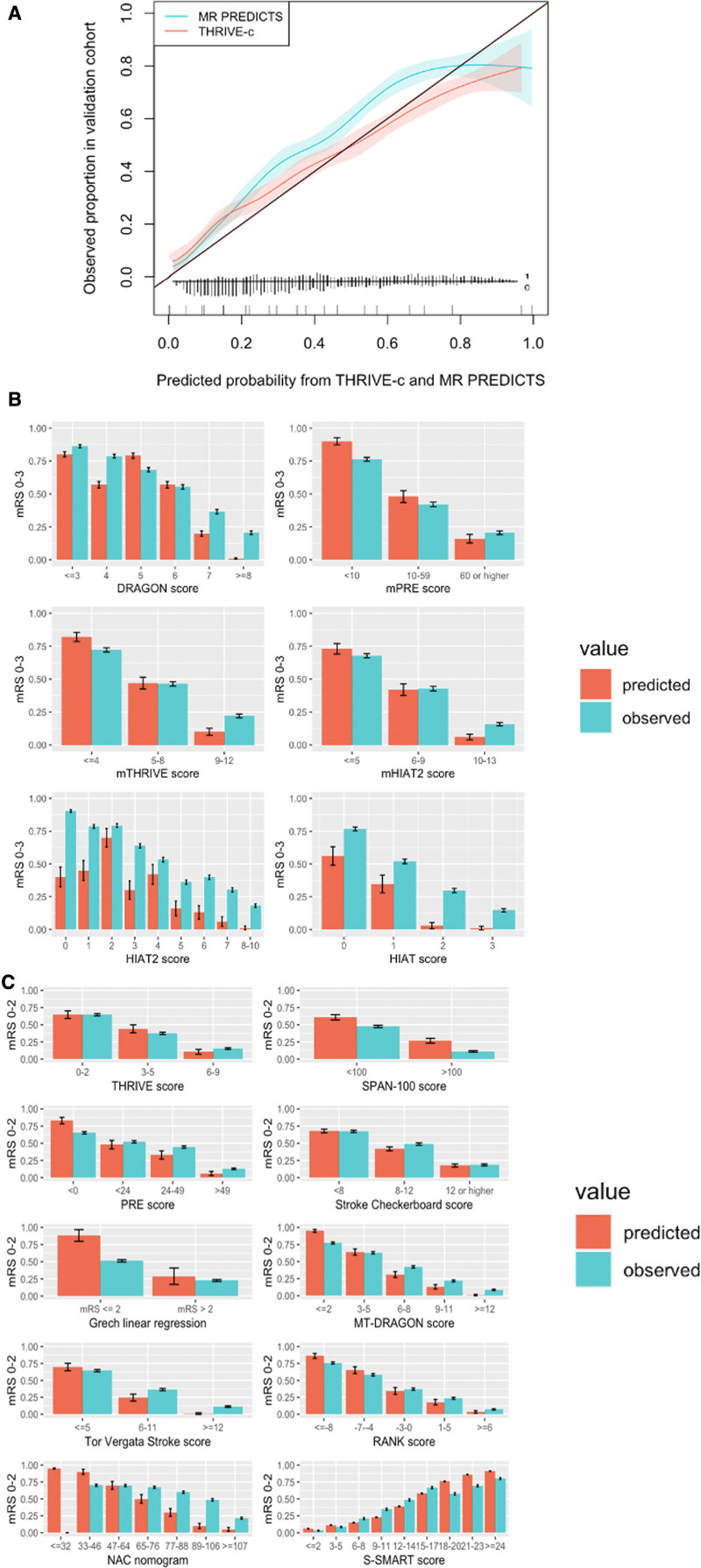
**Predicted vs observed proportion of good functional outcome measured by the modified Rankin Scale (mRS) for included models.** **A**, Models that predicted outcomes with logistic regression (MR PREDICTS and THRIVE-c). **B**, Risk scores with a calculation of points for certain risk categories with accompanying risks of good functional outcome (%) for mRS score 0–3. **C**, For mRS score 0–2. For risk scores, the predicted vs observed proportions of patients with a good functional outcome were analyzed since no calibration graph could be derived because the model output is not probabilistic.

The AUC ranged from 0.61 (SPAN-100 [Stroke Prognostication Using Age and NIH Stroke Scale]) to 0.80 (MR PREDICTS; Table 2, Figure 2).^8–10^ Multiple models showed similar performance in the upper range of discrimination. For models that predicted functional outcome with mRS cut points 0 to 2 (good) or 3 to 6 (poor), multiple models showed good to excellent discrimination, namely as the iScore (0.73 [95% CI, 0.72–0.75]), DRAGON score (0.73 [95% CI, 0.71–0.75]), the MT-DRAGON score (0.72 [95% CI, 0.70–0.74]), the S-SMART score (0.74 [95% CI, 0.72–0.75]), the THRIVE-c score (0.74 [95% CI, 0.72–0.75]), and MR PREDICTS (0.80 [95% CI, 0.78–0.81]). For models that predicted functional outcome with mRS cut points 0 to 3 (good) or 4 to 6 (poor), DRAGON (0.73 [95% CI, 0.71–0.75]), and HIAT ([Houston Intra-Arterial Therapy]; 0.71 [95% CI, 0.69–0.73]) showed best discriminative performance. Models that included patient comorbidities in their models generally had better discriminative performance than models without patient comorbidities in their models. No such trend in discriminative performance was observed for models with inclusion of radiological variables compared with models that did not include radiological variables (Figure 2). For models that predicted outcomes as mRS score of 0–2 (good) or mRS score 3–6 (poor), MR PREDICTS’ AUC was significantly different from other AUCs (*P*≤0.0001). The iScore, S-SMART, and THRIVE-c were also significantly different from other scores but did not differ significantly from each other (Table VII in the Supplemental Material). For models that predicted outcomes with mRS score 0–3 (good) or 4–6 (poor), DRAGON AUC was significantly higher than other models (Table VIII in the Supplemental Material). Calibration varied widely between models (Figure 3). The 5 models with the best-calibration intercept were the RANK scale (0.00 [95% CI, −0.08 to 0.09]), Stroke Checkerboard (−0.05 [95% CI, −0.13 to 0.03]), THRIVE score (−0.06 [95% CI, −0.14 to 0.02]), THRIVE-c score (0.08 [95% CI, −0.02 to 0.17]), and the mHIAT2 (−0.09 [95% CI, 0.00–0.16]). Models with the best-calibration slope were Stroke Checkerboard (0.97 [95% CI, 0.88–1.08]), MR PREDICTS (0.93 [95% CI, 0.85–1.01]), THRIVE (0.84 [95% CI, 0.75–0.95]), S-SMART (0.76 [95% CI, 0.69–0.86]), and the mHIAT2 (0.74 [95% CI, 0.65–0.83]). The best-calibrated models with a combination of a good intercept and slope were the Stroke Checkerboard, THRIVE, and the mHIAT2 (Table 2).

**Table 2.**
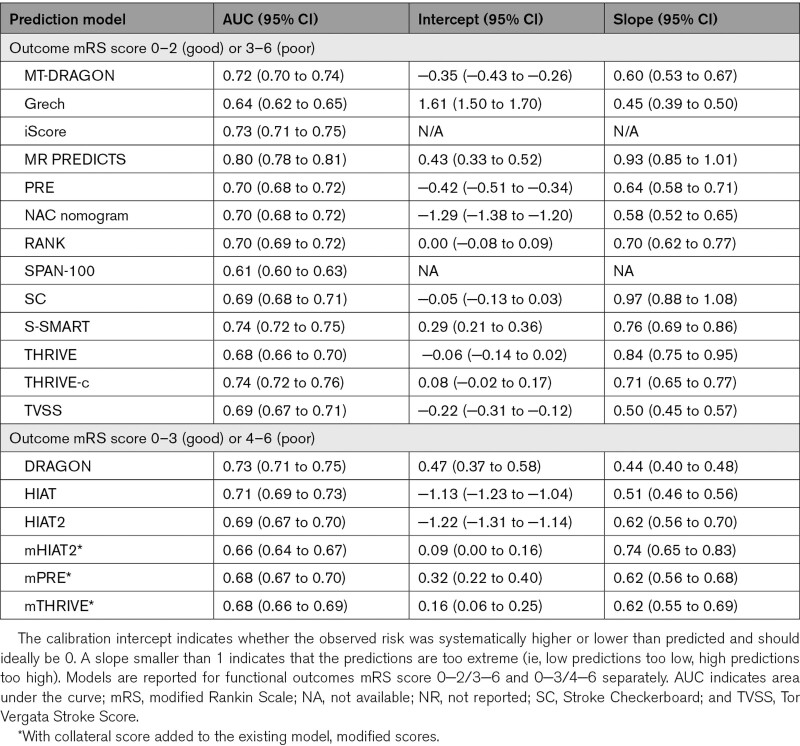
Discrimination (AUC) and Calibration (Intercept and Slope) per Included Model

The smallest average error (E_avg_) between predicted and observed probabilities was found in the Stroke Checkerboard score (1.5%). The largest E_avg_ was found in the model of Grech et al^22^ (25.7%). The median E_avg_ in all models was 8.2%, with a mean E_avg_ of 10.7%. The maximum absolute error (E_max_) varied from 1.8% (Stroke Checkerboard score) to 36.8% (Grech et al^22^). The median E_max_ was 13.5%, while the mean E_max_ was 16.6% (Table IX in the Supplemental Material).

## Discussion

We conducted a systematic literature search that identified 29 articles and 19 outcome prediction models for patients receiving EVT. Some of these models showed promising results for prediction of functional outcome after EVT, such as the THRIVE-c score, the S-SMART score, and MR PREDICTS. MR PREDICTS had the highest discriminative performance of all models assessed, while THRIVE-c combined good discriminative performance with less overprediction in calibration than MR PREDICTS. Other models also showed relatively good calibration, such as the Stroke Checkerboard score, the mHIAT2 score, and the THRIVE score, but demonstrated relatively poor discriminative performance.

A majority of models showed methodological shortcomings. Several studies excluded patients with missing values, which may have led to bias in patient selection and further analysis.^52^ Most models were not internally validated and did not correct for overfitting, leading to systematic overestimation or underestimation. In addition, almost no models were calibrated during development or in other external validation studies or were only assessed with a Hosmer-Lemeshow goodness-of-fit test. A Hosmer-Lemeshow test is not sufficient for assessment of calibration since power of the test increases with sample size and may reject a model with only slight deviation from the observed outcome. In addition, this test does not imply the direction of the misclassification in the model. It was, however, notable that some models with poor methodological quality did show good predictive performance, despite several shortcomings in the development of the model. A positive trend in reporting both discrimination and calibration was observed in more recent studies that described the development of the MT-DRAGON score, the S-SMART score, and MR PREDICTS. Researchers should be encouraged to be alert in the development of prediction models to avoid shortcomings in methodological quality and to be able to more accurately predict functional outcome for patients who suffer from an acute ischemic stroke. A large number of new prediction models has been published, with more newly developed models appearing each year. We encourage researchers to validate and recalibrate existing models so that existing models will be more reliable and could be better implemented in daily clinical practice.

Regarding presentation of published prediction models, risk scores that assigned points to values of a variable were often described. In some articles, multiple points in the risk scores were grouped together for prediction of outcome, leading to loss of information. Twenty-two of the 27 models evaluated for their model performance were described as such simplified risk scores. Therefore, the predicted risks may have oversimplified the more complicated real-life situation. Most risk scores included in this overview were developed as simplified versions to remain simple and easy to use, posing the tradeoff between complexity and prognostic accuracy of the model versus simplicity and uncomplicated use in situations that require urgent care. For most scores based on regression, application devices are available for an easier and more precise estimation of patient outcome in real clinical practice. Online calculators exist for the MR PREDICTS, the THRIVE score, the iScore, the DRAGON score, and the PRE score. Since many models had approximately equal predictive performance, the choice of model should be based on the preference of model simplicity and data availability in the clinical setting.

Prestroke mRS has proven to be an important and robust predictor of functional outcome in patients with ischemic stroke.^3,53^ We confirmed in our study that models that included prestroke mRS or other factors that described patient comorbidity showed a better predictive performance than models that did not include these factors. This may indicate that patient history may play a large role in determining functional outcome for EVT patients. However, many models mostly included patients with a low pre-mRS. In our validation cohort, we included a broad range of patients with all possible pre-mRS scores; patients with an mRS score of 4 or higher were sparse. High prestroke mRS values may be difficult to model, first of all because they are infrequent, and second because they could represent temporary disability. Prestroke mRS as a strong predictor should be nuanced and should be investigated further.

We did not observe a correlation with inclusion of radiological variables; however, this could be attributable to many other factors. Increasingly, patients with broader inclusion criteria are being investigated for EVT, such as patients with a lower Alberta Stroke Program Early CT Score and other occlusion sites. In our validation cohort, we did include patients with all possible Alberta Stroke Program Early CT Score, however, the number of patients with a low Alberta Stroke Program Early CT Score was small. Radiological variables may play a larger role in outcome prediction for these patients.

### Other Studies

The THRIVE score has been extensively validated. In earlier articles, the THRIVE score showed good predictive performance.^2,4,54^ However, its performance was comparable to other prediction models in this study. The THRIVE-c score was developed on a large patient cohort and has been validated for EVT patients.^47,48^ In our study, THRIVE-c score showed good predictive performance compared with other models. MR PREDICTS has been developed with data from the MR CLEAN trial, whereas validation of the included models has been performed with data from the MR CLEAN registry parts I and II. The patients in our validation cohort were treated in the same country and health care system as the MR CLEAN trial population that was used to develop MR PREDICTS. This correlation between the derivation cohort might explain the high predictive performance of MR PREDICTS. However, the inclusion criteria in the registry were broader than in the trial, and our cohort, therefore, consists of a patient population with more severe and more widely varying characteristics. In addition, MR PREDICTS also showed good discriminative power in other settings and is the only prediction model that predicted the ordinal mRS outcome, which illustrates a more valuable prediction.^49,55^ The iScore and DRAGON score were developed for patients receiving intravenous thrombolysis but showed good discrimination.^19,28^ The DRAGON score shows moderate calibration. Both scores have been developed on a large data set and have been internally validated during their development, demonstrating that even in patients undergoing EVT, discriminative power is higher compared with other scores developed for patients for EVT specifically.

### Limitations

There are several limitations that may have influenced the results in this study. Models that were included predicted different outcomes. Some models, such as the HIAT and DRAGON, had different cut points for good or poor functional outcome than other models. Therefore, we reported these models separately in our results. Models that predicted good or poor outcome with the same cut points were grouped together (such as mRS score 0–2 versus mRS score 3–6). It should, however, be emphasized that models that predict success (good outcome) and failure (poor outcome) may have different applications and goals when developed.

Many studies claim that their model can be used for treatment selection, however, most did not include patients with and without treatment in their development cohort. Only 2 models used treatment as a variable in their model (MR PREDICTS and S-SMART).^30,39^ Most models predict outcome after EVT but not treatment benefit. Even when the predicted outcome with treatment is moderate or poor, treatment can still be of added value to the patient, especially when the chance of a good outcome without treatment is very small. This is a major limitation of the investigated prediction models and should be taken into consideration when contemplating their use for clinical practice.

Some prediction tools that included radiological variables could not be validated. In our results, there was no clear distinction in discriminative performance between models with radiological variables and models without radiological variables. It is not yet certain whether radiological variables are of added value in predicting functional outcome. These variables have yet to be further investigated.^56^ All validated models included age and clinical severity (National Institutes of Health Stroke Scale/Canadian Neurological Score), and therefore, no claim could be made whether models performed better when these variables were included.

Many models did not describe which generation thrombectomy device was used in their patient cohort. This may have influenced the predictive performance when older devices were used for EVT.

Studies that were not in English were excluded, which may have led missing models which would have a good predictive performance in our validation cohort.

In addition, machine learning algorithms are a new and rapidly emerging method of predicting patient outcomes.^27,33,34,37,50^ Unfortunately, it was not possible to reproduce these predictive models in this study. Machine learning algorithms are difficult to validate since no reproducible model is available. This leads to problems for application in clinical practice. In addition, a machine learning algorithm is difficult to recalibrate and adapt to other populations and different clinical settings. This method of prediction is, however, promising and may be further investigated in future research, but does not yet prove to be superior over logistic regression models.^57^

A minor limitation of our approach is that there is currently no clear definition of what range of values constitute good intercepts and slopes for the calibration. We have tried to objectify calibration measures with a selection of the five models with the best intercept and slope. We acknowledge that further methodologic research is needed.

### Conclusions and Consequences for Clinical Practice

In conclusion, after a systematic search of published prediction models, we have externally validated and assessed published prediction models that estimate functional outcome (mRS) in patients with anterior circulation acute ischemic stroke eligible for EVT within 6.5 hours of onset. Many models have been developed but only few meet methodologic standards. A large number of models has been published, but not all models are equally useful for real-world implementation. The THRIVE-c and MR PREDICTS show the best combination of discrimination and calibration. Of these 2, the latter also predicts treatment benefit instead of merely outcome after treatment. Nevertheless, several other models have relatively good predictive performance as well, therefore, predictive performance should be one of several factors (eg, simplicity, data availability, and population similarity) to select the optimal model for real-world implementation.

## Article Information

### Acknowledgments

We are grateful to Elise Krabbendam, information specialist of the Medical Library of the Erasmus MC University Medical Center, Rotterdam, the Netherlands, for her help with the systematic literature search. For full details of the acknowledgements of the MR CLEAN (Multicenter Randomized Clinical Trial of Endovascular Treatment for Acute Ischemic Stroke in the Netherlands) registry, see the Appendix in the Supplemental Material.

### Sources of Funding

The MR CLEAN (Multicenter Randomized Clinical Trial of Endovascular Treatment for Acute Ischemic Stroke in the Netherlands) registry is partially funded by unrestricted grants from Toegepast Wetenschappelijk Instituut voor Neuromodulatie, Twente University (Twin), Erasmus MC, AMC, and MUMC.

### Disclosures

Dr Dippel reports funding from the Dutch Heart Foundation, Brain Foundation Netherlands, The Netherlands Organisation for Health Research and Development, Health Holland Top Sector Life Sciences & Health, and unrestricted grants from Penumbra Inc, Stryker European Operations BV, Medtronic, Thrombolytic Science, LLC, and Cerenovus for research, all paid to institution. Dr Majoie reports grants from CVON/Dutch Heart Foundation, grants from European Commission, grants from Dutch Health Evaluation Program, grants from Stryker, and grants from TWIN Foundation outside this submitted work; and shareholder of Nico-lab. Dr van der Lugt reports grants from Stryker, grants from Medtronic, grants from Penumbra, grants from Cerenovus, grants from Thrombolytic science Inc, grants from Siemens, and grants from GE healthcare outside this submitted work. Dr Burke reports grants from NIH outside this submitted work. The other authors report no conflicts.

### Supplemental Materials

Supplemental Material I–IV

Supplemental Figure I

Supplemental Tables I–IX

Supplemental Appendix

## Supplementary Material



## References

[1] Boehme AK Rawal PV Lyerly MJ Albright KC Bavarsad Shahripour R Palazzo P Kapoor N Alvi M Houston JT Harrigan MR. Investigating the utility of previously developed prediction scores in acute ischemic stroke patients in the stroke belt. J Stroke Cerebrovasc Dis. 2014;23:2001–2006. doi: 10.1016/j.jstrokecerebrovasdis.2014.02.003 25113079 10.1016/j.jstrokecerebrovasdis.2014.02.003 PMC4780244

[2] Ishkanian AA McCullough-Hicks ME Appelboom G Piazza MA Hwang BY Bruce SS Hannan LM Connolly ES Lavine SD Meyers PM. Improving patient selection for endovascular treatment of acute cerebral ischemia: a review of the literature and an external validation of the Houston IAT and THRIVE predictive scoring systems. Neurosurg Focus. 2011;30:E7. doi: 10.3171/2011.3.FOCUS1144 10.3171/2011.3.FOCUS1144 21631231

[3] Raza SA Rangaraju S. A review of pre-intervention prognostic scores for early prognostication and patient selection in endovascular management of large vessel occlusion stroke. Interv Neurol. 2018;7:171–181. doi: 10.1159/000486539 29719555 10.1159/000486539 PMC5920952

[4] De La Ossa NP Rangaraju S Jovin T Davalos A. Validation of predictive scales of functional outcome after endovascular therapy using the revascat data. Conference start. 2016;47:AWP26.

[5] Moher D Liberati A Tetzlaff J Altman DG; PRISMA Group. Preferred reporting items for systematic reviews and meta-analyses: the PRISMA statement. PLoS Med. 2009;6:e1000097. doi: 10.1371/journal.pmed.1000097 19621072 10.1371/journal.pmed.1000097 PMC2707599

[6] Wolff RF Moons KGM Riley RD Whiting PF Westwood M Collins GS Reitsma JB Kleijnen J Mallett S; PROBAST Group†. PROBAST: a tool to assess the risk of bias and applicability of prediction model studies. Ann Intern Med. 2019;170:51–58. doi: 10.7326/M18-1376 30596875 10.7326/M18-1376

[7] Tan IY Demchuk AM Hopyan J Zhang L Gladstone D Wong K Martin M Symons SP Fox AJ Aviv RI. CT angiography clot burden score and collateral score: correlation with clinical and radiologic outcomes in acute middle cerebral artery infarct. AJNR Am J Neuroradiol. 2009;30:525–531. doi: 10.3174/ajnr.A1408 19147716 10.3174/ajnr.A1408 PMC7051470

[8] DW Hosmer LS. Applied Logistic Regression. 2000. 2nd ed. John Wiley and Sons; 160–164.

[9] Metz CE. Basic principles of ROC analysis. Semin Nucl Med. 1978;8:283–298. doi: 10.1016/s0001-2998(78)80014-2 112681 10.1016/s0001-2998(78)80014-2

[10] Larner A. Dementia in Clinical Practice: A Neurological Perspective. Pragmatic Studies in the Cognitive Function Clinic. 2014. 2nd ed. Springer

[11] DeLong ER DeLong DM Clarke-Pearson DL. Comparing the areas under two or more correlated receiver operating characteristic curves: a nonparametric approach. Biometrics. 1988;44:837–845. 3203132

[12] Steyerberg EW Vergouwe Y. Towards better clinical prediction models: seven steps for development and an ABCD for validation. Eur Heart J. 2014;35:1925–1931. doi: 10.1093/eurheartj/ehu207 24898551 10.1093/eurheartj/ehu207 PMC4155437

[13] Steyerberg EW. Clinical Prediction Models: A Practical Approach to Development, Validation and Updating. 2009. Springer

[14] Van Calster B Nieboer D Vergouwe Y De Cock B Pencina MJ Steyerberg EW. A calibration hierarchy for risk models was defined: from utopia to empirical data. J Clin Epidemiol. 2016;74:167–176. doi: 10.1016/j.jclinepi.2015.12.005 26772608 10.1016/j.jclinepi.2015.12.005

[15] Stevens RJ Poppe KK. Validation of clinical prediction models: what does the “calibration slope” really measure? J Clin Epidemiol. 2020;118:93–99. doi: 10.1016/j.jclinepi.2019.09.016 31605731 10.1016/j.jclinepi.2019.09.016

[16] Harrell FE. Regression Modeling Strategies: With Applications to Linear Models, Logistic Regression, and Survival Analysis. 2015. 2nd ed. Springer-Verlag

[17] Di Giuliano F Picchi E Sallustio F Ferrazzoli V Alemseged F Greco L Minosse S Da Ros V Diomedi M Garaci F Marziali S Floris R. Accuracy of advanced CT imaging in prediction of functional outcome after endovascular treatment in patients with large-vessel occlusion. Neuroradiol J. 2019;32:62–70. doi: 10.1177/1971400918805710 30303448 10.1177/1971400918805710 PMC6327363

[18] Wang A Pednekar N Lehrer R Todo A Sahni R Marks S Stiefel MF. DRAGON score predicts functional outcomes in acute ischemic stroke patients receiving both intravenous tissue plasminogen activator and endovascular therapy. Surg Neurol Int. 2017;8:149. doi: 10.4103/2152-7806.210993 28791192 10.4103/2152-7806.210993 PMC5525460

[19] Strbian D Meretoja A Ahlhelm FJ Pitkäniemi J Lyrer P Kaste M Engelter S Tatlisumak T. Predicting outcome of IV thrombolysis-treated ischemic stroke patients: the DRAGON score. Neurology. 2012;78:427–432. doi: 10.1212/WNL.0b013e318245d2a9 22311929 10.1212/WNL.0b013e318245d2a9

[20] Turc G Apoil M Naggara O Calvet D Lamy C Tataru AM Méder JF Mas JL Baron JC Oppenheim C Touzé E. Magnetic Resonance Imaging-DRAGON score: 3-month outcome prediction after intravenous thrombolysis for anterior circulation stroke. Stroke. 2013;44:1323–1328. doi: 10.1161/STROKEAHA.111.000127 23482603 10.1161/STROKEAHA.111.000127

[21] Ben Hassen W Raynaud N Bricout N Boulouis G Legrand L Ferrigno M Kazemi A Bretzner M Soize S Farhat W. MT-DRAGON score for outcome prediction in acute ischemic stroke treated by mechanical thrombectomy within 8 hours. J Neurointerv Surg. 2020;12:246–251. doi: 10.1136/neurintsurg-2019-015105 31427503 10.1136/neurintsurg-2019-015105

[22] Grech R Galvin PL Power S O’Hare A Looby S Brennan P Thornton J. Outcome prediction in acute stroke patients considered for endovascular treatment: a novel tool. Interv Neuroradiol. 2014;20:312–324. doi: 10.15274/INR-2014-10029 24976094 10.15274/INR-2014-10029 PMC4178769

[23] Hallevi H Barreto AD Liebeskind DS Morales MM Martin-Schild SB Abraham AT Gadia J Saver JL Grotta JC Savitz SI; UCLA Intra-Arterial Therapy Investigators. Identifying patients at high risk for poor outcome after intra-arterial therapy for acute ischemic stroke. Stroke. 2009;40:1780–1785. doi: 10.1161/STROKEAHA.108.535146 19359652 10.1161/STROKEAHA.108.535146 PMC4138312

[24] Sarraj A Albright K Barreto AD Boehme AK Sitton CW Choi J Lutzker SL Sun CH Bibars W Nguyen CB. Optimizing prediction scores for poor outcome after intra-arterial therapy in anterior circulation acute ischemic stroke. Stroke. 2013;44:3324–3330. doi: 10.1161/STROKEAHA.113.001050 23929748 10.1161/STROKEAHA.113.001050 PMC4135710

[25] Peng F Zheng W Li F Wang J Liu Z Chen X Xiao L Sun W Liu X. Elevated mean platelet volume is associated with poor outcome after mechanical thrombectomy. J Neurointerv Surg. 2018;10:25–28. doi: 10.1136/neurintsurg-2016-012849 28082446 10.1136/neurintsurg-2016-012849

[26] Ryu CW Kim BM Kim HG Heo JH Nam HS Kim DJ Kim YD. Optimizing outcome prediction scores in patients undergoing endovascular thrombectomy for large vessel occlusions using collateral grade on computed tomography angiography. Neurosurgery. 2019;85:350–358. doi: 10.1093/neuros/nyy316 30010973 10.1093/neuros/nyy316

[27] Hilbert A Ramos LA van Os HJA Olabarriaga SD Tolhuisen ML Wermer MJH Barros RS van der Schaaf I Dippel D Roos YBWEM. Data-efficient deep learning of radiological image data for outcome prediction after endovascular treatment of patients with acute ischemic stroke. Comput Biol Med. 2019;115:103516. doi: 10.1016/j.compbiomed.2019.103516 31707199 10.1016/j.compbiomed.2019.103516

[28] Saposnik G Kapral MK Liu Y Hall R O’Donnell M Raptis S Tu JV Mamdani M Austin PC; Investigators of the Registry of the Canadian Stroke Network; Stroke Outcomes Research Canada (SORCan) Working Group. IScore: a risk score to predict death early after hospitalization for an acute ischemic stroke. Circulation. 2011;123:739–749. doi: 10.1161/CIRCULATIONAHA.110.983353 21300951 10.1161/CIRCULATIONAHA.110.983353

[29] Flint AC Xiang B Gupta R Nogueira RG Lutsep HL Jovin TG Albers GW Liebeskind DS Sanossian N Smith WS; TREVO-2 Trialists. THRIVE score predicts outcomes with a third-generation endovascular stroke treatment device in the TREVO-2 trial. Stroke. 2013;44:3370–3375. doi: 10.1161/STROKEAHA.113.002796 24072003 10.1161/STROKEAHA.113.002796 PMC4157913

[30] Venema E Mulder MJHL Roozenbeek B Broderick JP Yeatts SD Khatri P Berkhemer OA Emmer BJ Roos YBWEM Majoie CBLM. Selection of patients for intra-arterial treatment for acute ischaemic stroke: development and validation of a clinical decision tool in two randomised trials. BMJ. 2017;357:j1710. doi: 10.1136/bmj.j1710 28468840 10.1136/bmj.j1710 PMC5418887

[31] Li X Zou Y Hu J Li XM Huang CP Shan YJ Nyame L Zhao Z Sun C Ibrahim M. A NAC nomogram to predict the probability of three-month unfavorable outcome in Chinese acute ischemic stroke patients treated with mechanical thrombectomy. Int J Neurosci. 2021;131:163–169. doi: 10.1080/00207454.2020.1733565 32083963 10.1080/00207454.2020.1733565

[32] Fargen KM Chaudry I Turner RD Bennett JA Turk A Mocco J. A novel clinical and imaging based score for predicting outcome prior to endovascular treatment of acute ischemic stroke. J Neurointerv Surg. 2013;5 suppl 1 i38–i43. doi: 10.1136/neurintsurg-2012-010513 23144081 10.1136/neurintsurg-2012-010513

[33] Nishi H Oishi N Ishii A Ono I Ogura T Sunohara T Chihara H Fukumitsu R Okawa M Yamana N. Predicting clinical outcomes of large vessel occlusion before mechanical thrombectomy using machine learning. Stroke. 2019;50:2379–2388. doi: 10.1161/STROKEAHA.119.025411 31409267 10.1161/STROKEAHA.119.025411

[34] Nishi H Oishi N Ishii A Ono I Ogura T Sunohara T Chihara H Fukumitsu R Okawa M Yamana N. Deep learning-derived high-level neuroimaging features predict clinical outcomes for large vessel occlusion. Stroke. 2020;51:1484–1492. doi: 10.1161/STROKEAHA.119.028101 32248769 10.1161/STROKEAHA.119.028101

[35] Rangaraju S Aghaebrahim A Streib C Sun CH Ribo M Muchada M Nogueira R Frankel M Gupta R Jadhav A. Pittsburgh Response to Endovascular therapy (PRE) score: optimizing patient selection for endovascular therapy for large vessel occlusion strokes. J Neurointerv Surg. 2015;7:783–788. doi: 10.1136/neurintsurg-2014-011351 25320054 10.1136/neurintsurg-2014-011351

[36] Xiong Y Wang H Zhang M Zhou Z Wang W Liu X Fisher M. Risk stratification for endovascular treatment in acute anterior circulation occlusive stroke. J Stroke Cerebrovasc Dis. 2019;28:104442. doi: 10.1016/j.jstrokecerebrovasdis.2019.104442 31627996 10.1016/j.jstrokecerebrovasdis.2019.104442

[37] Raoult H Lassalle MV Parat B Rousseau C Eugène F Vannier S Evain S Le Bras A Ronziere T Ferre JC. DWI-based algorithm to predict disability in patients treated with thrombectomy for acute stroke. AJNR Am J Neuroradiol. 2020;41:274–279. doi: 10.3174/ajnr.A6379 32001446 10.3174/ajnr.A6379 PMC7015200

[38] Liggins JT Yoo AJ Mishra NK Wheeler HM Straka M Leslie-Mazwi TM Chaudhry ZA Kemp S Mlynash M Bammer R. A score based on age and DWI volume predicts poor outcome following endovascular treatment for acute ischemic stroke. Int J Stroke. 2015;10:705–709. doi: 10.1111/ijs.12207 24207136 10.1111/ijs.12207 PMC4048330

[39] Kim TJ Lee JS Oh MS Kim JW Yoon JS Lim JS. Predicting functional outcome based on linked data after acute ischemic stroke: S-SMART Score. Transl Stroke Res. 2020;11:1296–1305. doi: 10.1007/s12975-020-00815-y 32306239 10.1007/s12975-020-00815-y

[40] Łasocha B Brzegowy P Słowik A Latacz P Pułyk R Popiela TJ. Outcome estimation based on multimodal computed tomography examination in acute ischaemic stroke patients treated with mechanical thrombectomy. Wideochir Inne Tech Maloinwazyjne. 2019;14:560–566. doi: 10.5114/wiitm.2019.84761 31908703 10.5114/wiitm.2019.84761 PMC6939209

[41] Espinosa de Rueda M Parrilla G Manzano-Fernández S García-Villalba B Zamarro J Hernández-Fernández F. Combined multimodal computed tomography score correlates with futile recanalization after thrombectomy in patients with acute stroke. Stroke. 2015;46:2517–2522. doi: 10.1161/STROKEAHA.114.008598 26219650 10.1161/STROKEAHA.114.008598

[42] Song D Lee K Kim EH Kim YD Kim J Song TJ Lee HS Nam HS Heo JH. Value of utilizing both ASPECTS and CT angiography collateral score for outcome prediction in acute ischemic stroke. Int J Stroke. 2015;10:1018–1023. doi: 10.1111/ijs.12505 25907633 10.1111/ijs.12505

[43] Almekhlafi MA Davalos A Bonafe A Chapot R Gralla J Pereira VM Goyal M; STAR Registry Investigators. Impact of age and baseline NIHSS scores on clinical outcomes in the mechanical thrombectomy using solitaire FR in acute ischemic stroke study. AJNR Am J Neuroradiol. 2014;35:1337–1340. doi: 10.3174/ajnr.A3855 24557701 10.3174/ajnr.A3855 PMC7966577

[44] Saposnik G Guzik AK Reeves M Ovbiagele B Johnston SC. Stroke Prognostication using age and NIH stroke scale: SPAN-100. Neurology. 2013;80:21–28. doi: 10.1212/WNL.0b013e31827b1ace 23175723 10.1212/WNL.0b013e31827b1ace PMC3589202

[45] Le Bouc R Clarençon F Meseguer E Lapergue B Consoli A Turc G Naggara O Duong DL Servan J Reiner P. Efficacy of endovascular therapy in acute ischemic stroke depends on age and clinical severity. Stroke. 2018;49:1686–1694. doi: 10.1161/STROKEAHA.117.020511 29915120 10.1161/STROKEAHA.117.020511

[46] Flint AC Cullen SP Faigeles BS Rao VA. Predicting long-term outcome after endovascular stroke treatment: the totaled health risks in vascular events score. AJNR Am J Neuroradiol. 2010;31:1192–1196. doi: 10.3174/ajnr.A2050 20223889 10.3174/ajnr.A2050 PMC7965456

[47] Flint AC Rao VA Chan SL Cullen SP Faigeles BS Smith WS Bath PM Wahlgren N Ahmed N Donnan GA. Improved ischemic stroke outcome prediction using model estimation of outcome probability: the THRIVE-c calculation. Int J Stroke. 2015;10:815–821. doi: 10.1111/ijs.12529 26045081 10.1111/ijs.12529

[48] Kastrup A Brunner F Hildebrandt H Roth C Winterhalter M Gießing C Papanagiotou P. THRIVE score predicts clinical and radiological outcome after endovascular therapy or thrombolysis in patients with anterior circulation stroke in everyday clinical practice. Eur J Neurol. 2017;24:1032–1039. doi: 10.1111/ene.13328 28556351 10.1111/ene.13328

[49] Sallustio F Toschi N Mascolo AP Marrama F Morosetti D Da Ros V Gandini R Alemseged F Koch G Diomedi M. Selection of anterior circulation acute stroke patients for mechanical thrombectomy. J Neurol. 2019;266:2620–2628. doi: 10.1007/s00415-019-09454-2 31270665 10.1007/s00415-019-09454-2

[50] Van Os HJA Ramos LA Hilbert A Van Leeuwen M Van Walderveen MAA Kruyt ND. Predicting outcome of endovascular treatment for acute ischemic stroke: potential value of machine learning algorithms. Front Neurol. 2018;9:784. doi: 10.3389/fneur.2018.00784 30319525 10.3389/fneur.2018.00784 PMC6167479

[51] Wu X Liu G Zhou W Ou A Liu X Wang Y Zhou S Luo W Liu B. Outcome prediction for patients with anterior circulation acute ischemic stroke following endovascular treatment: a single-center study. Exp Ther Med. 2019;18:3869–3876. doi: 10.3892/etm.2019.8054 31641377 10.3892/etm.2019.8054 PMC6796376

[52] Sterne JA White IR Carlin JB Spratt M Royston P Kenward MG Wood AM Carpenter JR. Multiple imputation for missing data in epidemiological and clinical research: potential and pitfalls. BMJ. 2009;338:b2393. doi: 10.1136/bmj.b2393 19564179 10.1136/bmj.b2393 PMC2714692

[53] Quinn TJ Taylor-Rowan M Coyte A Clark AB Musgrave SD Metcalf AK Day DJ Bachmann MO Warburton EA Potter JF. Pre-stroke modified Rankin scale: evaluation of validity, prognostic accuracy, and association with treatment. Front Neurol. 2017;8:275. doi: 10.3389/fneur.2017.00275 28659859 10.3389/fneur.2017.00275 PMC5468801

[54] Matsumoto K Nohara Y Soejima H Yonehara T Nakashima N Kamouchi M. Stroke prognostic scores and data-driven prediction of clinical outcomes after acute ischemic stroke. Stroke. 2020;51:1477–1483. doi: 10.1161/STROKEAHA.119.027300 32208843 10.1161/STROKEAHA.119.027300

[55] Venema E Roozenbeek B Mulder MJHL Brown S Majoie CBLM Steyerberg EW Demchuk AM Muir KW Dávalos A Mitchell PJ. Prediction of outcome and endovascular treatment benefit: validation and update of the MR PREDICTS decision tool. Stroke. 2021;52:2764–2772. doi: 10.1161/STROKEAHA.120.032935 34266308 10.1161/STROKEAHA.120.032935 PMC8378416

[56] Soliman F Gupta A Delgado D Kamel H Pandya A. The role of imaging in clinical stroke scales that predict functional outcome: a systematic review. Neurohospitalist. 2017;7:169–178. doi: 10.1177/1941874417708128 28974995 10.1177/1941874417708128 PMC5613872

[57] Gravesteijn BY Nieboer D Ercole A Lingsma HF Nelson D van Calster B Steyerberg EW; CENTER-TBI Collaborators. Machine learning algorithms performed no better than regression models for prognostication in traumatic brain injury. J Clin Epidemiol. 2020;122:95–107. doi: 10.1016/j.jclinepi.2020.03.005 32201256 10.1016/j.jclinepi.2020.03.005

