# Supplementary material for: Outcome Prediction Models for Endovascular Treatment of Ischemic Stroke: Systematic Review and External Validation
Source: Stroke. 2021 Nov 4;53(3):825–36. doi: 10.1161/STROKEAHA.120.033445 (PMC8884132; doi:10.1161/STROKEAHA.120.033445)
Supplement: Supplementary file 1 [file str-53-0825-s001.pdf]

SUPPLEMENTARY MATERIAL

Outcome Prediction Models For Patients Undergoing Endovascular Treatment For Acute Ischemic Stroke: A Systematic Review And External Validation

Supplementary Table I. PRISMA statement, information flow through the systematic search process.

Section/topic	#	Checklist item	Reported on page #
**TITLE**			
Title	1	Identify the report as a systematic review, meta-analysis, or both.	1
**ABSTRACT**			
Structured summary	2	Provide a structured summary including, as applicable: background; objectives; data sources; study eligibility criteria, participants, and interventions; study appraisal and synthesis methods; results; limitations; conclusions and implications of key findings; systematic review registration number.	2
**INTRODUCTION**			
Rationale	3	Describe the rationale for the review in the context of what is already known.	4
Objectives	4	Provide an explicit statement of questions being addressed with reference to participants, interventions, comparisons, outcomes, and study design (PICOS).	4
**METHODS**			
Protocol and registration	5	Indicate if a review protocol exists, if and where it can be accessed (e.g., Web address), and, if available, provide registration information including registration number.	4,5
Eligibility criteria	6	Specify study characteristics (e.g., PICOS, length of follow-up) and report characteristics (e.g., years considered, language, publication status) used as criteria for eligibility, giving rationale.	5
Information sources	7	Describe all information sources (e.g., databases with dates of coverage, contact with study authors to identify additional studies) in the search and date last searched.	4,5
Search	8	Present full electronic search strategy for at least one database, including any limits used, such that it could be repeated.	Suppl. I
Study selection	9	State the process for selecting studies (i.e., screening, eligibility, included in systematic review, and, if applicable, included in the meta-analysis).	4,5
Data collection process	10	Describe method of data extraction from reports (e.g., piloted forms, independently, in duplicate) and any processes for obtaining and confirming data from investigators.	4,5
Data items	11	List and define all variables for which data were sought (e.g., PICOS, funding sources) and any assumptions and simplifications made.	5
Risk of bias in individual studies	12	Describe methods used for assessing risk of bias of individual studies (including specification of whether this was done at the study or outcome level), and how this information is to be used in any data synthesis.	5
Summary measures	13	State the principal summary measures (e.g., risk ratio, difference in means).	6,7
Synthesis of results	14	Describe the methods of handling data and combining results of studies, if done, including measures of consistency (e.g., I^2) for each meta-analysis.	6,7
Risk of bias across studies	15	Specify any assessment of risk of bias that may affect the cumulative evidence (e.g., publication bias, selective reporting within studies).	5
Additional analyses	16	Describe methods of additional analyses (e.g., sensitivity or subgroup analyses, meta-regression), if done, indicating which were pre-specified.	6
**RESULTS**			
Study selection	17	Give numbers of studies screened, assessed for eligibility, and included in the review, with reasons for exclusions at each stage, ideally with a flow diagram.	7
Study characteristics	18	For each study, present characteristics for which data were extracted (e.g., study size, PICOS, follow-up period) and provide the citations.	7,8
Risk of bias within studies	19	Present data on risk of bias of each study and, if available, any outcome level assessment (see item 12).	8
Results of individual studies	20	For all outcomes considered (benefits or harms), present, for each study: (a) simple summary data for each intervention group (b) effect estimates and confidence intervals, ideally with a forest plot.	8,9
Synthesis of results	21	Present results of each meta-analysis done, including confidence intervals and measures of consistency.	8,9
Risk of bias across studies	22	Present results of any assessment of risk of bias across studies (see Item 15).	8
Additional analysis	23	Give results of additional analyses, if done (e.g., sensitivity or subgroup analyses, meta-regression [see Item 16]).	8,9
**DISCUSSION**			
Summary of evidence	24	Summarize the main findings including the strength of evidence for each main outcome; consider their relevance to key groups (e.g., healthcare providers, users, and policy makers).	9-12
Limitations	25	Discuss limitations at study and outcome level (e.g., risk of bias), and at review-level (e.g., incomplete retrieval of identified research, reporting bias).	13
Conclusions	26	Provide a general interpretation of the results in the context of other evidence, and implications for future research.	14
**FUNDING**			
Funding	27	Describe sources of funding for the systematic review and other support (e.g., supply of data); role of funders for the systematic review.	15

Supplementary Material I. Seach strategy conducted on 18th of May, 2020

Embase
('thrombectomy'/exp OR ('endovascular surgery'/de AND 'brain ischemia'/exp) OR 'embolectomy'/exp OR 'thrombectomy device'/exp OR 'embolectomy system'/de OR (EVT OR embolect* OR thrombect* OR Soehendra OR Solitaire OR Trevo OR Penumbra OR AngioJet OR APERIO OR ASPIRE OR BONnet OR ((CRC OR pREset OR Revive OR Catch) NEAR/3 (device* OR LITE)) OR ERIC OR FlowTriever OR MindFrame-Capture OR Rotarex OR MERCI OR Phenox-Clot OR ((stent*) NEAR/3 (retriever*)) OR ((thrombus* OR thrombi* OR embol*) NEAR/3 (aspirat* OR excision* OR remov*)) OR ((thrombolys* OR therap* OR treatment* OR procedure*) NEAR/6 (intra-arterial*)) OR ((endovascular* OR endo-vascular*) NEAR/12 (ischemi* OR ischaemi*))):ab,ti) **AND** ('prediction and forecasting'/exp OR 'prognostic assessment'/exp OR 'prognosis'/de OR (predict* OR forecast* OR prognos*):ab,ti) **AND** ('brain infarction'/exp OR 'cerebrovascular accident'/exp OR (CVA OR stroke* OR ((cerebr* OR brain* OR cerebellum* OR migrain* OR cortical* OR hemispher*) NEAR/3 (infarct*)) OR ((cerebr* OR brain* OR cerebellum*) NEAR/3 (accident* OR lesion* OR vasculopath* OR insult* OR attack* OR disturbance* OR apoplexy* OR apoplec* OR insuffic* OR arrest* OR failure* OR injur*))):ab,ti) *NOT ([animals]/lim NOT [humans]/lim)* NOT ([Conference Abstract]/lim)

Medline
(exp Thrombectomy/ OR (Endovascular Procedures/ AND exp Brain Ischemia/) OR exp Embolectomy/ OR (EVT OR embolect* OR thrombect* OR Soehendra OR Solitaire OR Trevo OR Penumbra OR AngioJet OR APERIO OR ASPIRE OR BONnet OR ((CRC OR pREset OR Revive OR Catch) ADJ3 (device* OR LITE)) OR ERIC OR FlowTriever OR MindFrame-Capture OR Rotarex OR MERCI OR Phenox-Clot OR ((stent*) ADJ3 (retriever*)) OR ((thrombus* OR thrombi* OR embol*) ADJ3 (aspirat* OR excision* OR remov*)) OR ((thrombolys* OR therap* OR treatment* OR procedure*) ADJ6 (intra-arterial*)) OR ((endovascular* OR endo-vascular*) ADJ12 (ischemi* OR ischaemi*))).ab,ti.) **AND** (exp Forecasting/ OR Prognosis/ OR (predict* OR forecast* OR prognos*).ab,ti.) **AND** (exp Brain Infarction/ OR exp Stroke/ OR (CVA OR stroke* OR ((cerebr* OR brain* OR cerebellum* OR migrain* OR cortical* OR hemispher*) ADJ3 (infarct*)) OR ((cerebr* OR brain* OR cerebellum*) ADJ3 (accident* OR lesion* OR vasculopath* OR insult* OR attack* OR disturbance* OR apoplexy* OR apoplec* OR insuffic* OR arrest* OR failure* OR injur*))).ab,ti.) *NOT (exp animals/ NOT humans/) NOT (news OR congres* OR abstract* OR book* OR chapter* OR dissertation abstract*).pt.*

Cochrane
((EVT OR embolect* OR thrombect* OR Soehendra OR Solitaire OR Trevo OR Penumbra OR AngioJet OR APERIO OR ASPIRE OR BONnet OR ((CRC OR pREset OR Revive OR Catch) NEAR/3 (device* OR LITE)) OR ERIC OR FlowTriever OR MindFrame-Capture OR Rotarex OR MERCI OR Phenox-Clot OR ((stent*) NEAR/3 (retriever*)) OR ((thrombus* OR thrombi* OR embol*) NEAR/3 (aspirat* OR excision* OR remov*)) OR ((thrombolys* OR therap* OR treatment* OR procedure*) NEAR/6 (((intra) NEXT/1 (arterial*)))) OR ((endovascular* OR ((endo) NEXT/1 (vascular*))) NEAR/12 (ischemi* OR ischaemi*))):ab,ti) **AND** ((predict* OR forecast* OR prognos*):ab,ti) **AND** ((CVA OR stroke* OR ((cerebr* OR brain* OR cerebellum* OR migrain* OR cortical* OR hemispher*) NEAR/3 (infarct*)) OR ((cerebr* OR brain* OR cerebellum*) NEAR/3 (accident* OR lesion* OR vasculopath* OR insult* OR attack* OR disturbance* OR apoplexy* OR apoplec* OR insuffic* OR arrest* OR failure* OR injur*))):ab,ti)

Web of Science

TS=(((EVT OR embolect* OR thrombect* OR Soehendra OR Solitaire OR Trevo OR Penumbra OR AngioJet OR APERIO OR ASPIRE OR BONnet OR ((CRC OR pREset OR Revive OR Catch) NEAR/2 (device* OR LITE)) OR ERIC OR FlowTriever OR MindFrame-Capture OR Rotarex OR MERCI OR Phenox-Clot OR ((stent*) NEAR/2 (retriever*)) OR ((thrombus* OR thrombi* OR embol*) NEAR/2 (aspirat* OR excision* OR remov*)) OR ((thrombolys* OR therap* OR treatment* OR procedure*) NEAR/5 (intra-arterial*)) OR ((endovascular* OR endo-vascular*) NEAR/12 (ischemi* OR ischaemi*)))) **AND** ((predict* OR forecast* OR prognos*)) **AND** ((CVA OR stroke* OR ((cerebr* OR brain* OR cerebellum* OR migrain* OR cortical* OR hemispher*) NEAR/2 (infarct*)) OR ((cerebr* OR brain* OR cerebellum*) NEAR/2 (accident* OR lesion* OR vasculopath* OR insult* OR attack* OR disturbance* OR apoplexy* OR apoplec* OR insuffic* OR arrest* OR failure* OR injur*)))) *NOT ((animal* OR rat OR rats OR mouse OR mice OR murine OR dog OR dogs OR canine OR cat OR cats OR feline OR rabbit OR cow OR cows OR bovine OR rodent* OR sheep OR ovine OR pig OR swine OR porcine OR veterinar* OR chick* OR zebrafish* OR baboon* OR nonhuman* OR primate* OR cattle* OR goose OR geese OR duck OR macaque* OR avian* OR bird* OR fish*) NOT (human* OR patient* OR women OR woman OR men OR man))*) *AND DT=(Article OR Review)*

Google Scholar – (random top-200)
embolectomy|thrombectomy|"stent retriever"|"thrombus|thrombi|embolus aspiration|excision|remove"|"endovascular|endo-vascular ischemia|ischaemia" "predict|forecast|prediction|prognosis" stroke|"cerebrum|brain infarct|attack|injury"

Database	*# of refs*	*After de-dup*
Embase	2385	2339
Medline	1825	262
Web of Science	2148	619
Cochrane	289	140
Google Scholar (random top-200)	200	108
**Total**	**6847**	**3468**

Supplementary Material II. PROBAST questionnaire

DOMAIN 1: Participants

A. Risk of Bias

Describe the sources of data and criteria for participant selection:
1.1 Were appropriate data sources used, e.g. cohort, RCT or nested case-control study data?
1.2 Were all inclusions and exclusions of participants appropriate?
Risk of bias introduced by selection of participants RISK: (low/ high/ unclear)
Rationale of bias rating:

B. Applicability

Describe included participants, setting and dates:
Concern that the included participants and setting do not match the review question:
CONCERN: (low/ high/ unclear)
Rationale of applicability rating:

DOMAIN 2: Predictors

A. Risk of Bias

List and describe predictors included in the final model, e.g. definition and timing of assessment:
2.1 Were predictors defined and assessed in a similar way for all participants?
2.2 Were predictor assessments made without knowledge of outcome data?
2.3 Are all predictors available at the time the model is intended to be used?
Risk of bias introduced by predictors or their assessment RISK: (low/ high/ unclear)
Rationale of bias rating:

B. Applicability

Concern that the definition, assessment or timing of predictors in the model do not match the review question:
CONCERN: (low/ high/ unclear)
Rationale of applicability rating:

DOMAIN 3: Outcome

A. Risk of Bias

Describe the outcome, how it was defined and determined, and the time interval between predictor assessment and outcome determination:
3.1 Was the outcome determined appropriately?
3.2 Was a pre-specified or standard outcome definition used?
3.3 Were predictors excluded from the outcome definition?
3.4 Was the outcome defined and determined in a similar way for all participants?
3.5 Was the outcome determined without knowledge of predictor information?
3.6 Was the time interval between predictor assessment and outcome determination appropriate?
Risk of bias introduced by the outcome or its determination RISK: (low/ high/ unclear)
Rationale of bias rating:

B. Applicability

At what time point was the outcome determined:
If a composite outcome was used, describe the relative frequency/distribution of each contributing outcome:

Concern that the outcome, its definition, timing or determination do not match the review question:
CONCERN: (low/ high/ unclear)
Rationale of applicability rating:

DOMAIN 4: Analysis
Risk of Bias
Describe numbers of participants, number of candidate predictors, outcome events and events per candidate predictor:

Describe how the model was developed (for example in regards to modelling technique (e.g. survival or logistic modelling), predictor selection, and risk group definition):

Describe whether and how the model was validated, either internally (e.g. bootstrapping, cross validation, random split sample) or externally (e.g. temporal validation, geographical validation, different setting, different type of participants):

Describe the performance measures of the model, e.g. (re)calibration, discrimination, (re)classification, net benefit, and whether they were adjusted for optimism:

Describe any participants who were excluded from the analysis:

Describe missing data on predictors and outcomes as well as methods used for missing data:

4.1 Were there a reasonable number of participants with the outcome?
4.2 Were continuous and categorical predictors handled appropriately?
4.3 Were all enrolled participants included in the analysis?
4.4 Were participants with missing data handled appropriately?
4.5 Was selection of predictors based on univariable analysis avoided?
4.6 Were complexities in the data (e.g. censoring, competing risks, sampling of controls) accounted for appropriately?
4.7 Were relevant model performance measures evaluated appropriately?
4.8 Were model overfitting and optimism in model performance accounted for?
4.9 Do predictors and their assigned weights in the final model correspond to the results from multivariable analysis?
Risk of bias introduced by the analysis RISK: (low/ high/ unclear)
Rationale of bias rating:

Model	Di Guilano[17]	DRAGON[18, 19]	MT-DRAGON[20, 21]	Grech[22]	HIAT[23]	HIAT2[24]	HIAT-MPV[25]	mHIAT2[26]	iScore[28, 29]	MR PREDICTS[30]	NAC[31]	NAV[32]	PRE[35]	mPRE[26]	RANK[36]	SAD[38]	S-SMART[39]	SMCTS[40, 41]	Song et al.[42]	SPAN-100[43, 44]	SC[45]	THRIVE[46]	mTHRIVE[26]*	THRIVE-c[47, 48]	TVSS[49]	Wu et al.[51]	Total
**Clinical Characteristics**																											
Age		•	•	•	•	•	•	•	•	•	•	•	•	•	•	•	•			•	•	•	•	•	•	•	23
Stroke severity*		•	•	•	•	•	•	•	•	•	•	•	•	•	•		•			•	•	•	•	•	•	•	22
IVT treatment			•							•							•										3
Onset to treatment time		•	•							•																	3
Sex									•								•									•	3
Stroke mechanism									•								•										2
Onset to arrival ER															•												1
Onset - reperfusion time																										•	1
Systolic blood pressure										•																	1
**Laboratory Characteristics**																											
Blood glucose		•	•		•	•	•	•	•						•										•	•	10
Creatinine											•																1
Mean platelet volume							•																				1
**Patient Comorbidities**																											
Pre disability mRS		•	•						•	•							•								•		6
Diabetes mellitus									•	•												•	•	•			5
Atrial fibrillation									•													•	•	•			4
Hypertension																						•	•	•			3
Smoking									•																		1
Stroke										•																	1
CAD, CHF									•																		1
Cancer, dementia, renal dialysis									•																		1
**Radiological Characteristics**																											
Collaterals	•			•				•		•				•	•			•	•				•		•		10
ASPECTS			•			•		•		•			•	•	•				•						•		9
Occlusion location			•							•																	2
Cerebral blood volume												•						•									2
DWI lesion volume																•										•	2
Clot burden score																									•		1
Time to maximum tissue residue**	•																										1
Cerebral blood flow	•																										1
Hypodensity/dense MCA sign on CT		•																									1
Contrast enhancement																		•									1
Total variables included in the model	3	6	8	3	3	4	4	5	11	11	3	2	3	4	6	2	6	3	2	2	2	5	6	5	7	6	

Supplementary Table II. Overview of pre-intervention variables used in different models for prediction of outcome after acute endovascular treatment for ischemic stroke.

* Stroke severity measured by NIHSS/CNS
** Measured in $T_{max,v}^{16-25s}$
SC = Stroke Checkerboard score, TVSS = Tor Vergata Stroke Score, ER = Emergency department, CAD = coronary artery disease, CHF = congestive heart faliure.
mHIAT2, mPRE en mTHRIVE are the regular prediction models with collateral score added to the model.
Onset to treatment time is defined as time of onset to EVT groin puncture. When a model used time to IVT treatment, the score was adapted to time to EVT treatment.

The columns in grey represent the models that could not be validated since some variables were not included in the validation cohort.

Supplementary Table III. Overview of calculation per included model

Model	Author	Developed for patients treated with:	Outcome**	Variable categories	Points	Probability of outcome
**DRAGON**	Strbian et al. (2012)[19] / Wang et al. (2017)[18]	IVT	mRS 0-3	No (hyper)dense cerebral artery sign or early infarct signs	0	≤ 3 = 80%<br>4 = 57%<br>5 = 79%<br>6 =57%<br>7 = 20%<br>≥ 8 = 1%
				Either a (hyper)dense cerebral artery sign or early infarct signs	1	
				Both a (hyper)dense cerebral artery sign and early infarct signs	2	
				Prestroke mRS ≤ 1	0	
				Prestroke mRS >1	1	
				Age < 65	0	
				Age 65-79	1	
				Age ≥ 80	2	
				Glucose ≤ 8 mmol/L	0	
				Glucose ≥ 8 mmol/L	1	
				Onset to treatment time ≤ 90 min	0	
				Onset to treatment time > 90 min	1	
				NIHSS 0-4	0	
				NIHSS 5-9	1	
				NIHSS 10-15	2	
				NIHSS > 15	3	
				**Total score**	**0-10**	https://www.mdcalc.com/dragon-score-post-tpa-stroke-outcome
**MT-DRAGON**	Ben Hassen et al.[20, 21]	EVT	mRS 4-6	Onset to groin puncture time ≤ 4.5h	0	≤2 = 5%,<br>3-5 = 36%,<br>6-8 = 69%,<br>9-11 = 87%<br>12-14 = 99%<br>15-20 = 99%
				Onset to groin puncture time > 4.5h	4	
				No prior IVT	2	
				Prior IVT	0	
				Prestroke mRS ≤1	0	
				Prestroke mRS >1	4	
				Age 65 -79	1	
				Age ≥ 80	2	
				Glucose ≤ 8 mmol/L,	0	
				Glucose ≥ 8 mmol/L	2	
				Occlusion site (internal carotid ±M1, tandem)	2	
				NIHSS 0-4	0	
				NIHSS 5-9	1	

				NIHSS 10-15	2	
				NIHSS > 15	3	
				**Total score**	**0-20**	
**HIAT**	Hallevi et al. (2009)[23]	EVT	mRS 4-6	Age > 75	1	0 = 44%<br>1 = 65%<br>2 = 97%,<br>3 = 99%
				NIHSS > 18	1	
				Glucose > 150 mg/dL	1	
				**Total score**	**0-3**	
**HIAT2**	Sarraj et al. (2013)[24]	EVT	mRS 4-6	Age ≤ 59	0	0 = 60%<br>1 = 55%<br>2 = 30%<br>3 = 70%<br>4 = 58%<br>5 = 84%<br>6 = 87%<br>7 = 94%<br>8-10 = 99%
				Age 60-79	2	
				Age ≥ 80	4	
				Glucose < 150 mg/dL	0	
				Glucose ≥ 150 mg/dL	1	
				NIHSS ≤ 10	0	
				NIHSS 11- 20	1	
				NIHSS ≥ 21	2	
				ASPECTS 8-10	0	
				ASPECTS ≤ 7	3	
				**Total score**	**0-10**	
**iScore**	Saposnik et al. (2011)[28] / Flint et al. (2013)[29]	IVT	mRS 0-2	Age	Age (in years)	
				Sex, female	0	*N/A*
				Sex, male	10	
				CNS*** = 0	105	
				CNS ≤ 4	65	
				CNS 5-7	40	
				CNS ≥ 8	0	
				Stroke subtype, lacunar	0	
				Stroke subtype, nonlacunar	30	
				Stroke subtype, undetermined origin	35	
				Risk factor, atrial fibrillation	10	
				Risk factor, congestive heart failure	10	
				Comorbid condition, cancer	10	
				Comorbid condition, renal dialysis	35	
				Preadmission disability, independent	0	
				Preadmission disability, dependent	15	
				Glucose < 7.5 mmol/L	0	
				Glucose ≥ 7.5 mmol/L	15	http://www.sorcan.ca/iscore/
				**Total score**	**0-245**	
**mHIAT2, mTHRIVE, mPRE**	Ryu et al. (2019)[26]	EVT	mRS 4-6	HIAT2 + collateral score, good	0	mHIAT2:<br>≤ 5 = 27%<br>6-9 = 58%<br>≥ 10 = 94%
				HIAT2 + collateral score, poor	3	

				THRIVE + collateral score, good	0	mTHRIVE:<br>≤ 4 = 18%<br>5-8 = 53%<br>≥ 9 = 90%<br><br>mPRE:<br>≤ 9 = 10%<br>10 - 60 = 52%<br>≥ 61 = 84%
				THRIVE + collateral score, poor	3	
				PRE + collateral score, good	0	
				PRE + collateral score, poor	10	
				**Total score**	**Original scores + collateral values**	
**RANK scale**	Xiong et al.[36]	EVT	mRS 4-6	Age < 50	0	≤ -8 = 14%<br>-7 - -4 = 35%<br>-3 - 0 = 66%<br>1 – 5 = 82%<br>≥ 5 = 97%
				Age 50-59	1	
				Age 60-69	3	
				Age 70-79	4	
				Age 80-89	5	
				Time of onset to presentation to emergency department <120	0	
				120 -179	1	
				180 - 299	2	
				≥ 300	3	
				Blood glucose ≤ 8.6 mmol/L	0	
				Blood glucose > 8.6 ss mmol/L	3	
				NIHSS ≤ 16	0	
				NIHSS > 16	3	
				ASPECTS ≤ 7	0	
				ASPECTS > 7	-4	
				Collateral status 0	0	
				Collateral status 1	-3	
				Collateral status 2	-6	
				Collateral status 3	-7	
				**Total**	**-11 - 14**	
**S-SMART**	Kim et al.[39]	EVT ± IVT	mRS 0-2	NIHSS 0-7	12	≤ 2 = 16%<br>3-5 = 11%<br>6-8 = 15%<br>9-11 = 23%<br>12-14 = 39%<br>15-17 = 58%<br>18-20 = 76%<br>21-23 = 86%<br>≥ 24 = 91%
				NIHSS 8-13	4	
				NIHSS ≥ 14	0	
				Sex, male	2	
				Sex, female	0	
				Stroke mechanism, SVO	2	
				Stroke mechanism, CE	1	
				Stroke mechanism, other	0	
				Age < 80	4	
				Prestroke mRS = 0	4	
				Thrombolysis/thrombectomy treatment, no	0	
				Thrombolysis/thrombectomy treatment, IVT only	1	
				Thrombolysis/thrombectomy treatment, EVT only	1	
				Thrombolysis/thrombectomy	3	

				treatment, IVT + EVT		
				**Total score**	**0 - 34**	
**SPAN-100**	Saposnik et al. (2013)[44] / Almekhlafi et al. (2014)[43]	IVT	mRS 0-2	Age	Age in years	< 100 = 61%<br>≥ 100 = 27%
				NIHSS	Total NIHSS	
				**Total score**	**Continuous**	
**Stroke Checkerboard**	Le Bouc et al. (2018)[45]	EVT	mRS 0-2	Age, per decade, start from 0 (< 50, 50-59, 60-69, 70-79, 80- 89, ≥ 90)	1	≤ 7 = 68%<br>8-11 = 42%<br>≥ 12 = 18%
				NIHSS, per 5 points, start from 2 (0-5, 6-10, 11-15, 16-20, ≥ 21)	2	
				**Total score**	**2-15**	
**THRIVE**	Flint et al. (2010)[46]	EVT	mRS 0-2	NIHSS ≤ 10	0	0 -2 = 65%<br>3-5 = 44%<br>6-9 = 11%
				NIHSS 11-20	2	
				NIHSS ≥ 21	4	
				Age ≤ 59	0	
				Age 60-79	1	
				Age ≥ 80	2	
				CDS* = 0	0	
				CDS* = 1	1	
				CDS* = 2	2	
				CDS* = 3	3	
				**Total score**	**0-9**	
**Tor Vergata Stroke Score**	Sallustio et al.[49]	EVT	mRS 4-6	Age > 80	2	0-5 = 03.2%<br>6-11 = 75.2%<br>12-18 = 99%
				Prestroke mRS > 0	3	
				ASPECTS ≤ 8	2	
				Poor collateral flow	3	
				NIHSS > 17	2	
				Clot Burden Score ≤5	3	
				Glucose > 111 mg/dL****	3	
				**Total score**	**0 - 18**	

* CDS: 1 point for either a history of hypertension, diabetes mellitus, or atrial fibrillation
**As reported in validation article
***CNS: stroke severity measured by the Canadian Neurological Scale
**** Glucose was converted to mmol/L for analysis
Probabilities of outcome and corresponding grouping of points were extracted from the articles developed or validated for patients receiving endovascular therapy. Probabilities of 1 or 0 were adapted to probabilities of 0.99 and 0.01, since patients with a probability of 0 or 1 would be excluded from analysis.
SVO = small vessel occlusion, CE = cardioembolic.
Collateral scores were derived from either CTA or DSA. Depending on the prediction model, different methods for derivation of the collateral grading system were used (Poor/good; 0/1/2/3, where a higher grade stands for better collaterals). Collateral scores were implemented for validation as graded in the validation cohort [7] .

Supplementary Table IV. Regression models

Author	Model	Developed for:	Outcome**	Equation	Probability
Flint et al. (2015)[47]/ Kastrup et al. (2017)[48]	**THRIVE-c**	IVT / EVT	mRS 0-2	-4.94 – 0.035*age – 0.19*NIHSS – 0.11*CDS1 – 0.41*CDS2 – 0.70*CDS3	Transformation to probability from log odds*
Grech et al. (2014)[22]	**Grech**	EVT	mRS 3-6	– 0.729 0.049*age + 0.071*NIHSS – 1.456*collaterals	≤ 2 = 0.12, >2 = 0.71
Li et al.[31]	**NAC nomogram**	EVT	mRS 3-6	2.5*NIHSS+ 0.39*creatinine+ 0.33*age	≤ 32 = 0.05, 33-46 = 0.1, 47-64 = 0.3, 65-76 = 0.5, 77-88 = 0.7, 89-106 = 0.9, >106 = 0.95
Rangaraju et al. (2015)[35]	**PRE**	EVT	mRS 0-2	Age + 2*NIHSS – 10*ASPECTS https://www.mdcalc.com/pittsburgh-response-endovascular-therapy-pre-score	≤ 0 = 0.83, 0-24 = 0.48, 25-49 = 0.33, > 49= 0.06
Venema et al. (2017)[30]	**MR PREDICTS**	EVT	mRS 0-2	0.85 * EVT - 0.0041 * age - 0.000029 * pmax(age-47,0)^3 + 0.000071 * pmax(age-66.95,0)^3 - 0.000042 * pmax(age - 80.86,0)^3 - 0.068 * NIHSS - 0.38 * premRS - 0.49 * previous diabetes - 0.00054 * SBP - 0.0000029 * pmax(SBP - 117,0)^3 + 0.0000052 *pmax(SBP - 144,0)^3 - 0.0000022 * pmax(SBP - 180,0)^3 + 0.53 * IVT + 0.11 * ASPECTS + 0.52 * (location=="M1") + 0.82 * (location=="M2") + 0.39 * collaterals - 0.11 * previous stroke - 0.0029 * time to groin - 0.60 * EVT * previous stroke - 0.0016 * EVT * time to groin + 0.12 * EVT * collaterals - 0.98 (https://mrpredicts.shinyapps.io/RRRR_1/)	Transformation to probability from log odds *

*Transformation with: $P = \frac{1}{1+e^{-\log(odds)}}$

Supplementary Material III. Models with variables that were not available in the validation cohort

Some variables used in selected prediction models were not available in the validation cohort and could therefore not be assessed on predictive performance. The NAV score[32], the SAD score[38], the SMCTS score[40], the model of Wu et al. [51] and the model of Di Giuliano et al.[17] described models with radiological characteristics that were not available in the validation cohort. These models included variables such as the time to maximum tissue residue ($\boldsymbol{T^{16-25s}_{max,v}}$)[17], cerebral blood flow (CBF)[17], cerebral blood volume (CBV)[32], and diffusion-weighted imaging (DWI) lesion volume[38]. The HIAT-MPV score[25] consisted of the HIAT score with an added variable, mean platelet volume (MPV) for improvement of the model, which was also not present in the validation cohort. The model of Song et al.[42] and the model of Raoult et al.[37] analyzed models with hierarchical classification and regression tree analysis and could not be externally validated with the MR CLEAN Registry.

Supplementary Table V. PROBAST quality assessment per prediction model

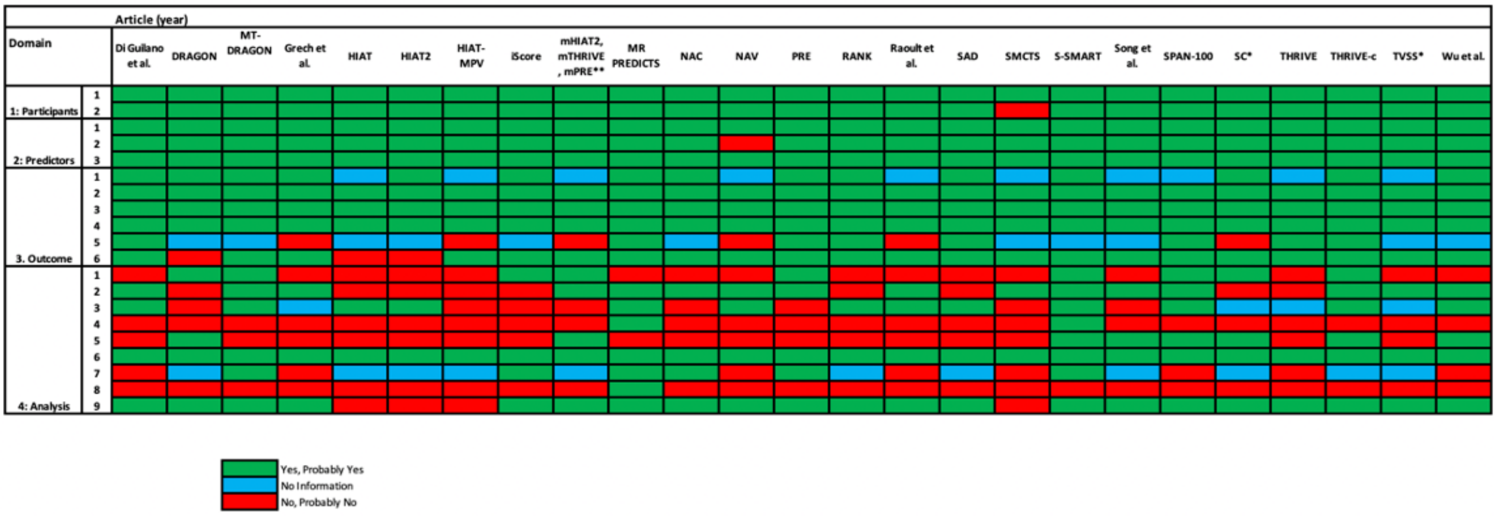

* SC = Stroke Checkerboard score, TVSS = Tor Vergata Stroke Score
** modified HIAT2, THRIVE and PRE scores with collaterals added to the existing models

Supplementary Material IV. Methodological analysis of included models

Only the DRAGON score, the MT-DRAGON score, the NAC nomogram, the RANK scale, the TVSS, the iScore, the THRIVE-c and the MR PREDICTS (31%) assessed predictive performance with internal validation. 12 out of 27 models (44%) were assessed for generalizability with external validation in either the article of model development or in other studies. External validation ranged from 1 to 34 validations available in literature (median [IQR] 6 [1.75, 13.50]). Only 1 model (MR PREDICTS) accounted for optimism and overfitting of the data. The S-SMART, MR PREDICTS, NAC nomogram and the MT-DRAGON scores were the only models that assessed calibration of their models in either a derivation or validation cohort. For 25 models (93%) a strategy to account for missing data was not used. Most of these models had been developed based on a complete-case analysis and the relation of missingness to the outcome had not been assessed. Nine studies that categorized continuous variables did not develop their cut-point values with empiric assessment of variable associations or based on prior theory. Instead, cut-point values seemed to have been arbitrarily chosen for variables included in the model.

Supplementary Figure I. Correlation between PROBAST methodological quality (number of questions answered with No/No information) and the Δ AUC (AUC reported for EVT in article of model development vs. AUC calculated in the MR CLEAN Registry validation cohort)

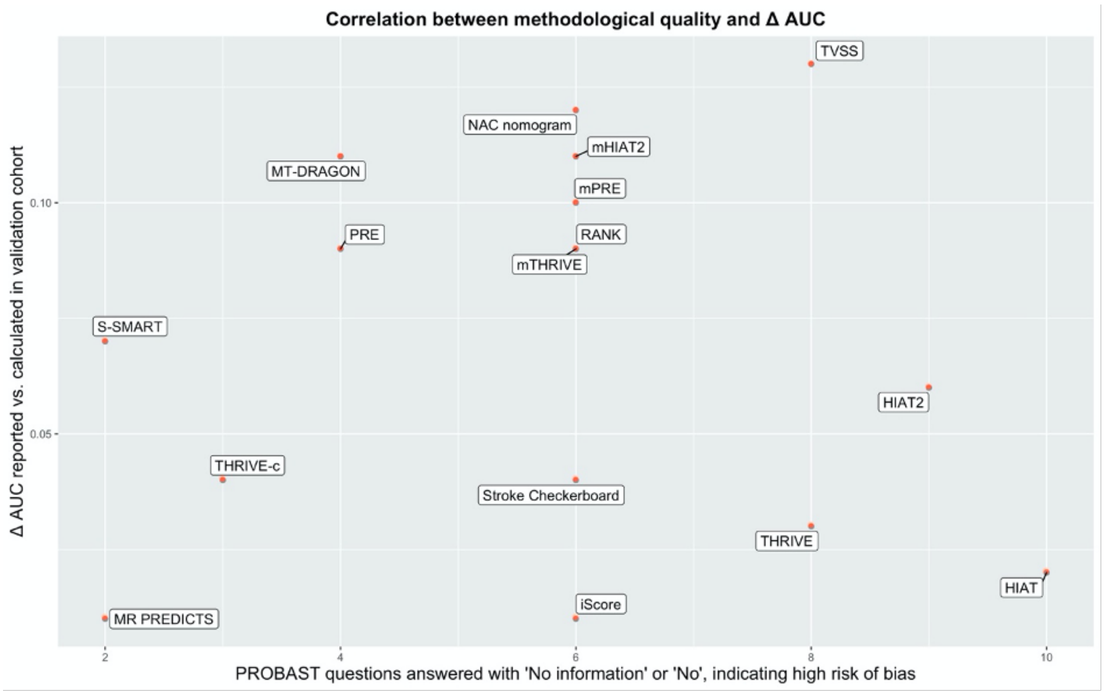

Supplementary Table VI. Baseline table

Variable	MR CLEAN Registry (n=3156)
Age, years (mean ± SD)	70.03 (14.15)
Glucose at baseline, mmol (mean (SD))	7.40 (2.47)
Systolic blood pressure, mmHg (mean (SD))	149.97 (24.78)
Diastolic blood pressure, mmHg (mean (SD))	82.33 (15.52)
Time to groin, minutes (mean (SD))	203.17 (73.04)
NIHSS (median [IQR])	16.00 [11.00, 19.00]
mRS (median [IQR])	3.00 [2.00, 6.00]
Pre-mRS (median [IQR])	0.00 [0.00, 1.00]
ASPECTS at baseline (median [IQR])	9.00 [7.00, 10.00]
Good functional outcome (mRS 0-2 (%))	1193 (40.5)
mRS 0-3 (HIAT classification of 'good outcome') (%)	1585 (53.8)
Diabetes (%)	502 (16.0)
Atrial fibrillation (%)	751 (24.1)
Hypertension (%)	1618 (52.4)
Previous MI (%)	438 (14.2)
Previous stroke (%)	527 (16.8)
Sex (%)	
**Male**	1640 (52.0)
**Female**	1516 (48.0)
Collaterals (%)	
**Absent**	186 (6.3)
**Filling <50% of occluded area**	1069 (36.1)
**>50% but < 100%**	1152 (38.9)
**100% of occluded area**	557 (18.8)
Smoking (%)	
**No**	1749 (55.9)
**Yes**	672 (21.5)
**Unknown**	707 (22.6)
IVT yes/no (%)	
**Yes**	2410 (76.5)
**No**	735 (23.3)
**Unknown**	7 (0.2)
Hyperdense artery sign on CT (%)	
**Yes**	1649 (54.4)
**No**	
Side of hyperdense artery sign on CT(%)	
**Left hemisphere**	797 (48.5)
**Right hemisphere**	847 (51.5)
Hyperdense artery sign location on CT (%)	
**ACI**	175 (10.8)
**MCA main stem**	1447 (89.2)
**ACA**	1 (0.1)
Occlusion segment (%)	
**Intracranial ICA**	155 (5.2)
**ICA-T**	640 (21.3)
**M1**	1764 (58.8)
**M2**	441 (14.7)

mRS at 90 days	
**0**	205
**1**	455
**2**	533
**3**	392
**4**	348
**5**	160
**6**	854
ASPECTS at baseline	
**0 - 4**	144
**5-7**	626
**8-10**	2282
Pre-mRS	
**0**	2093
**1**	408
**2**	230
**≥3**	355

		MT-DRAGON	Grech	iScore	MR PREDICTS	PRE	NAC	RANK	SPAN-100	SC	S-SMART	THRIVE	THRIVE-c	TVSS
**MT-DRAGON**	0.72 (0.70;0.74)	x												
**Grech**	0.64 (0.62;0.65)	<0.0001**	x											
**iScore**	0.73 (0.71;0.75)	0.28	<0.0001**	x										
**MR PREDICTS**	0.80 (0.78;0.81)	<0.0001**	<0.0001**	<0.0001**	x									
**PRE**	0.70 (0.68;0.72)	0.15	<0.0001**	0.00014**	<0.0001**	x								
**NAC**	0.70 (0.68;0.72)	0.045*	<0.0001**	0.0087*	<0.0001**	0.81	x							
**RANK**	0.70 (0.69;0.72)	0.086	<0.0001**	0.017*	<0.0001**	0.94	0.68	x						
**SPAN-100**	0.61 (0.60;0.63)	<0.0001**	0.006*	<0.0001**	<0.0001**	<0.0001**	<0.0001**	<0.0001**	x					
**SC**	0.69 (0.68;0.71)	0.04*	<0.0001**	<0.0001**	<0.0001**	0.34	0.74	0.51	<0.0001**	x				
**S-SMART**	0.74 (0.72;0.75)	0.12	<0.0001**	0.44	<0.0001**	<0.0001**	0.0018*	0.0037*	<0.0001**	<0.0001**	x			
**THRIVE**	0.68 (0.66;0.70)	0.0047*	0.00018**	<0.0001**	<0.0001**	0.055	0.29	0.16	<0.0001**	0.15	<0.0001**	x		
**THRIVE-c**	0.74 (0.72;0.76)	0.14	<0.0001**	0.27	<0.0001**	<0.0001**	0.0026*	0.0053*	<0.0001**	<0.0001**	0.915	<0.0001**	x	
**TVSS**	0.69 (0.67;0.71)	0.012*	<0.0001**	0.0024*	<0.0001**	0.61	0.76	0.4	<0.0001**	0.93	0.00035**	0.39	0.00058**	x

Supplementary Table VII. Discriminative performance of included outcome prediction models based on good outcome (mRS 0-2) or poor outcome (mRS 3-6) with + 95% confidence intervals and p-values for comparison of AUCs based on the DeLong test. p<0.05 = *, <0.001 =**. SC = Stroke Checkerboard, TVSS = Tor Vergata Stroke Score.

Supplementary Table VIII. Discriminative performance of included outcome prediction models based on good outcome (mRS 0-3) or poor outcome (mRS 4-6) with + 95% confidence intervals and p-values for comparison of AUCs based on the DeLong test. p<0.05 = *, <0.001 =**.
SC = Stroke Checkerboard, TVSS = Tor Vergata Stroke Score.

		DRAGON	HIAT	HIAT2	mHIAT2	mPRE	mTHRIVE
**DRAGON**	0.73 (0.71;0.75)	x					
**HIAT**	0.71 (0.69;0.73)	0.033*	x				
**HIAT2**	0.69 (0.67;0.70)	0.00038**	0.056	x			
**mHIAT2**	0.66 (0.64;0.67)	<0.0001**	<0.0001**	0.00047**	x		
**mPRE**	0.68 (0.67;0.70)	<0.0001**	0.0099*	0.53	0.0028*	x	
**mTHRIVE**	0.68 (0.66;0.69)	<0.0001**	0.0010**	0.26	0.028*	0.45	x

Supplementary Table IX. Estimated average (E_{avg}) and maximum error (E_{max}) between the calibrated models, describing the predicted probability of functional outcome and the loess-calibrated observed probabilities of functional outcome per prediction model.

Prediction model	E_{avg} (%)	E_{max} (%)
**DRAGON**	11.4	25.9
**MT-DRAGON**	7.9	13.3
**Grech**	25.7	36.8
**HIAT**	20.3	30.9
**HIAT2**	20.3	29.3
**mHIAT2****	3.4	6.7
**iScore**	20.4	30.4
**MR PREDICTS**	7.5	10.2
**PRE**	9.4	17.4
**mPRE****	6.6	13.6
**NAC**	19.8	24.7
**RANK**	5.5	10.2
**S-SMART**	8.4	8.2
**SPAN-100**	*N/A*	*N/A*
**SC***	1.5	1.8
**THRIVE**	4.6	5.5
**THRIVE-c**	4.9	8.4
**mTHRIVE****	5.8	10.8
**TVSS**	8.7	14.2

SC = Stroke Checkerboard score, TVSS = Tor Vergata Stroke Score
** modified HIAT2, THRIVE and PRE scores with collaterals added to the existing models

Supplementary Appendix. acknowledgements MR CLEAN Registry

Rotterdam, August 2019 - Registry part I+II

MR CLEAN Registry Investigators – group authors

Executive committee

Diederik W.J. Dippel[1];Aad van der Lugt[2];Charles B.L.M. Majoie[3];Yvo B.W.E.M. Roos[4];Robert J. van Oostenbrugge[5];Wim H. van Zwam[6];Jelis Boiten[14];Jan Albert Vos[8]

Study coordinators

Ivo G.H. Jansen[3];Maxim J.H.L. Mulder[1,2];Robert- Jan B. Goldhoorn[5,6];Kars C.J. Compagne[2];Manon Kappelhof[3];Josje Brouwer[4];Sanne J. den Hartog[1,2,40];Wouter H. Hinsenveld [5,6];

Local principal investigators

Diederik W.J. Dippel[1];Bob Roozenbeek[1];Aad van der Lugt[2];Adriaan C.G.M. van Es[2];Charles B.L.M. Majoie[3];Yvo B.W.E.M. Roos[4];Bart J. Emmer[3];Jonathan M. Coutinho[4];Wouter J. Schonewille[7];Jan Albert Vos[8]; Marieke J.H. Wermer[9];Marianne A.A. van Walderveen[10];Julie Staals[5];Robert J. van Oostenbrugge[5];Wim H. van Zwam[6];Jeannette Hofmeijer[11];Jasper M. Martens[12];Geert J. Lycklama à Nijeholt[13];Jelis Boiten[14];Sebastiaan F. de Bruijn[15];Lukas C. van Dijk[16];H. Bart van der Worp[17];Rob H. Lo[18];Ewoud J. van Dijk[19];Hieronymus D. Boogaarts[20];J. de Vries[22];Paul L.M. de Kort[21]; Julia van Tuijl[21] ; Jo P. Peluso[26];Puck Fransen[22];Jan S.P. van den Berg[22];Boudewijn A.A.M. van Hasselt[23];Leo A.M. Aerden[24];René J. Dallinga[25];Maarten Uyttenboogaart[28];Omid Eschgi[29];Reinoud P.H. Bokkers[29];Tobien H.C.M.L. Schreuder[30];Roel J.J. Heijboer[31];Koos Keizer[32];Lonneke S.F. Yo[33];Heleen M. den Hertog[22];Emiel J.C. Sturm[35]; Paul J.A.M. Brouwers[34]

Imaging assessment committee

Charles B.L.M. Majoie[3](chair);Wim H. van Zwam[6];Aad van der Lugt[2];Geert J. Lycklama à Nijeholt[13];Marianne A.A. van Walderveen[10];Marieke E.S. Sprengers[3];Sjoerd F.M. Jenniskens[27];René van den Berg[3];Albert J. Yoo[38];Ludo F.M. Beenen[3];Alida A. Postma[6];Stefan D. Roosendaal[3];Bas F.W. van der Kallen[13];Ido R. van den Wijngaard[13];Adriaan C.G.M. van Es[2];Bart J. Emmer[3];Jasper M. Martens[12]; Lonneke S.F. Yo[33];Jan Albert Vos[8]; Joost Bot[36], Pieter-Jan van Doormaal[2]; Anton Meijer[27];Elyas Ghariq[13]; Reinoud P.H. Bokkers[29];Marc P. van Proosdij[37];G. Menno Krietemeijer[33];Jo P. Peluso[26];Hieronymus D. Boogaarts[20];Rob Lo[18];Dick Gerrits[35];Wouter Dinkelaar[2]Auke P.A. Appelman[29];Bas Hammer[16];Sjoert Pegge[27];Anouk van der Hoorn[29];Saman Vinke[20].

Writing committee

Diederik W.J. Dippel[1](chair);Aad van der Lugt[2];Charles B.L.M. Majoie[3];Yvo B.W.E.M. Roos[4];Robert J. van Oostenbrugge[5];Wim H. van Zwam[6];Geert J. Lycklama à Nijeholt[13];Jelis Boiten[14];Jan Albert Vos[8];Wouter J. Schonewille[7];Jeannette Hofmeijer[11];Jasper M. Martens[12];H. Bart van der Worp[17];Rob H. Lo[18]

Adverse event committee

Robert J. van Oostenbrugge[5](chair);Jeannette Hofmeijer[11];H. Zwenneke Flach[23]

Trial methodologist

Hester F. Lingsma[40]

Research nurses / local trial coordinators

Naziha el Ghannouti[1];Martin Sterrenberg[1];Wilma Pellikaan[7];Rita Sprengers[4];Marjan Elfrink[11];Michelle Simons[11];Marjolein Vossers[12];Joke de Meris[14];Tamara Vermeulen[14];Annet Geerlings[19];Gina van Vemde[22];Tiny Simons[30];Gert Messchendorp[28];Nynke Nicolaij[28];Hester Bongenaar[32];Karin Bodde[24];Sandra Kleijn[34];Jasmijn Lodico[34]; Hanneke Droste[34];Maureen Wollaert[5];Sabrina Verheesen[5];D. Jeurrissen[5];Erna Bos[9];Yvonne Drabbe[15];Michelle Sandiman[15];Nicoline Aaldering[11];Berber Zweedijk[17];Jocova Vervoort[21];Eva Ponjee[22];Sharon Romviel[19];Karin Kanselaar[19];Denn Barning[10].

PhD / Medical students:

Esmee Venema[40]; Vicky Chalos[1,40]; Ralph R. Geuskens[3]; Tim van Straaten[19];Saliha Ergezen[1]; Roger R.M. Harmsma[1]; Daan Muijres[1]; Anouk de Jong[1];Olvert A. Berkhemer[1,3,6];Anna M.M. Boers[3,39]; J. Huguet[3];P.F.C. Groot[3];Marieke A. Mens[3];Katinka R. van Kranendonk[3];Kilian M. Treurniet[3];Manon L. Tolhuisen[3,39];Heitor Alves[3];Annick J. Weterings[3],Eleonora L.F. Kirkels[3],Eva J.H.F. Voogd[11];Lieve M. Schupp[3];Sabine L. Collette[28,29];Adrien E.D. Groot[4];Natalie E. LeCouffe[4];Praneeta R. Konduri[39];Haryadi Prasetya[39];Nerea Arrarte-Terreros[39];Lucas A. Ramos[39].

List of affiliations

Department of Neurology[1], Radiology[2], Public Health[40], Erasmus MC University Medical Center;
Department of Radiology and Nuclear Medicine[3], Neurology[4], Biomedical Engineering & Physics[39], Amsterdam UMC, University of Amsterdam, Amsterdam;
Department of Neurology[5], Radiology[6], Maastricht University Medical Center and Cardiovascular Research Institute Maastricht (CARIM);
Department of Neurology[7], Radiology[8], Sint Antonius Hospital, Nieuwegein;
Department of Neurology[9], Radiology[10], Leiden University Medical Center;
Department of Neurology[11], Radiology[12], Rijnstate Hospital, Arnhem;
Department of Radiology[13], Neurology[14], Haaglanden MC, the Hague;
Department of Neurology[15], Radiology[16], HAGA Hospital, the Hague;
Department of Neurology[17], Radiology[18], University Medical Center Utrecht;
Department of Neurology[19], Neurosurgery[20], Radiology[27], Radboud University Medical Center, Nijmegen;
Department of Neurology[21], Radiology[26], Elisabeth-TweeSteden ziekenhuis, Tilburg;
Department of Neurology[22], Radiology[23], Isala Klinieken, Zwolle;
Department of Neurology[24], Radiology[25], Reinier de Graaf Gasthuis, Delft;
Department of Neurology[28], Radiology[29], University Medical Center Groningen;
Department of Neurology[30], Radiology[31], Atrium Medical Center, Heerlen;
Department of Neurology[32], Radiology[33], Catharina Hospital, Eindhoven;
Department of Neurology[34], Radiology[35], Medical Spectrum Twente, Enschede;
Department of Radiology[36], Amsterdam UMC, Vrije Universiteit van Amsterdam, Amsterdam;
Department of Radiology[37], Noordwest Ziekenhuisgroep, Alkmaar;
Department of Radiology[38], Texas Stroke Institute, Texas, United States of America.
