# Supplementary figures and images for: Outcome Prediction Models for Endovascular Treatment of Ischemic Stroke: Systematic Review and External Validation
Source: Stroke. 2021 Nov 4;53(3):825–36. doi: 10.1161/STROKEAHA.120.033445 (PMC8884132; doi:10.1161/STROKEAHA.120.033445)

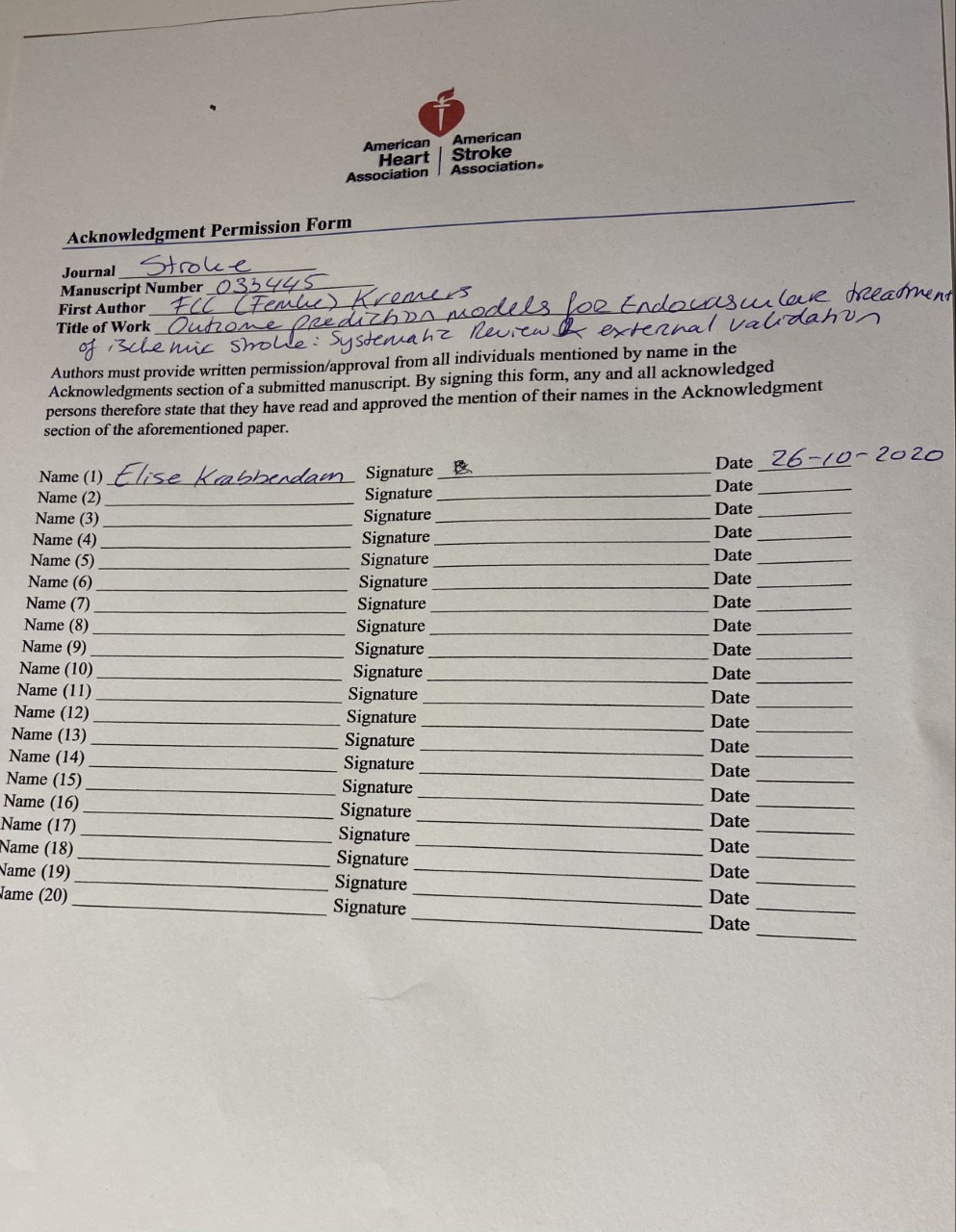

Supplement: Supplementary file 2 [file str-53-0825-s002.jpg]

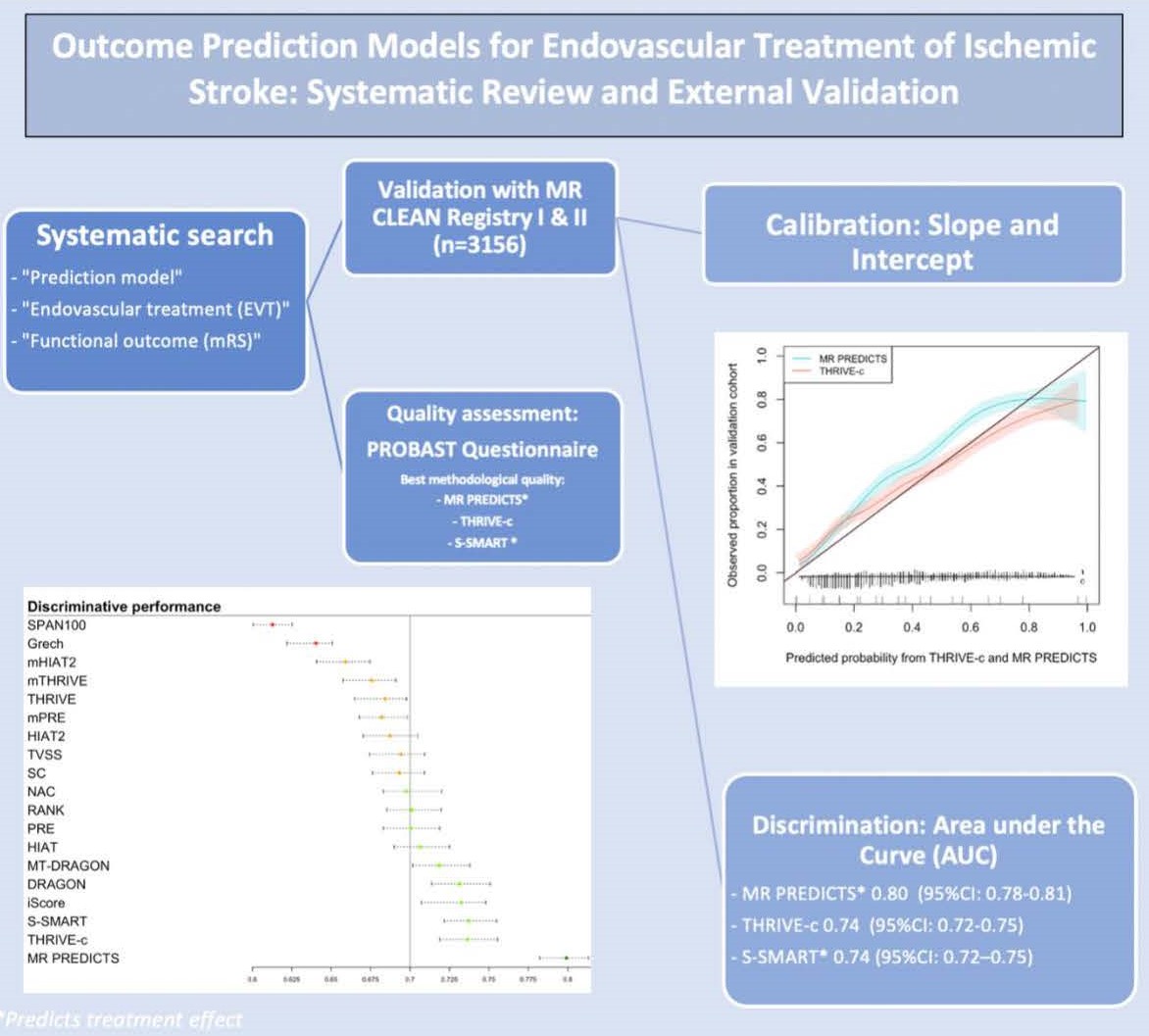

Supplement: Supplementary file 3 [file str-53-0825-s003.jpg]
